# Temporal progression of tau pathology and neuroinflammation in a rhesus monkey model of Alzheimer's disease

**DOI:** 10.1002/alz.13868

**Published:** 2024-06-21

**Authors:** Danielle Beckman, Giovanne B. Diniz, Sean Ott, Brad Hobson, Abhijit J. Chaudhari, Scott Muller, Yaping Chu, Akihiro Takano, Adam J. Schwarz, Chien‐Lin Yeh, Paul McQuade, Paramita Chakrabarty, Nicholas M. Kanaan, Maria S. Quinton, Arthur A. Simen, Jeffrey H. Kordower, John H. Morrison

**Affiliations:** ^1^ California National Primate Research Center University of California Davis Davis California USA; ^2^ Department of Radiology, School of Medicine University of California Davis Sacramento California USA; ^3^ ASU‐Banner Neurodegenerative Disease Research Center Arizona State University Tempe Arizona USA; ^4^ Takeda Development Center Americas, Inc. Lexington Massachusetts USA; ^5^ Department of Neuroscience, Center for Translational Research in Neurodegenerative Disease University of Florida Gainesville Florida USA; ^6^ Department of Translational Neuroscience, College of Human Medicine Michigan State University Grand Rapids Michigan USA; ^7^ Department of Neurological Sciences Rush University Medical Center Chicago Illinois USA; ^8^ Department of Neurology School of Medicine University of California Davis Sacramento California USA

**Keywords:** Alzheimer's disease, biomarkers, glial cells, nonhuman primates, tau

## Abstract

**INTRODUCTION:**

The understanding of the pathological events in Alzheimer's disease (AD) has advanced dramatically, but the successful translation from rodent models into efficient human therapies is still problematic.

**METHODS:**

To examine how tau pathology can develop in the primate brain, we injected 12 macaques with a dual tau mutation (P301L/S320F) into the entorhinal cortex (ERC). An investigation was performed using high‐resolution microscopy, magnetic resonance imaging (MRI), positron emission tomography (PET), and fluid biomarkers to determine the temporal progression of the pathology 3 and 6 months after the injection.

**RESULTS:**

Using quantitative microscopy targeting markers for neurodegeneration and neuroinflammation, as well as fluid and imaging biomarkers, we detailed the progression of misfolded tau spreading and the consequential inflammatory response induced by glial cells.

**DISCUSSION:**

By combining the analysis of several in vivo biomarkers with extensive brain microscopy analysis, we described the initial steps of misfolded tau spreading and neuroinflammation in a monkey model highly translatable to AD patients.

**Highlights:**

- Dual tau mutation delivery in the entorhinal cortex induces progressive tau pathology in rhesus macaques.
- Exogenous human 4R‐tau coaptates monkey 3R‐tau during transneuronal spread, in a prion‐like manner.
- Neuroinflammatory response is coordinated by microglia and astrocytes in response to tau pathology, with microglia targeting early tau pathology, while astrocytes engaged later in the progression, coincident with neuronal death.
- Monthly collection of CSF and plasma revealed a profile of changes in several AD core biomarkers, reflective of neurodegeneration and neuroinflammation as early as 1 month after injection.

## 1 BACKGROUND

Tau protein, the expression product of gene MAPT (microtubule‐associate protein tau), binds to tubulin with high affinity to regulate microtubule stability.^1^ In physiological conditions, tau is found predominantly in the axon, playing an important role in structural and functional axon integrity.^1^, ^2^ The abnormal processing of tau is associated with a wide range of neurodegenerative processes and is believed to play an essential role in the pathophysiology of Alzheimer's disease (AD).^2^, ^3^, ^4^ This abnormal processing is also associated with numerous tau post‐translational modifications in AD and other tauopathies,^5^ including hyperphosphorylation, truncation, misfolding, and the generation of insoluble tau aggregates, in addition to the translocation of tau to dendrites and the soma from the axonal compartment.^1^, ^2^ Tau fibrillization, in turn, often accompanies profound disruptions of the neuronal milieu, including glutamatergic neurotoxicity and an exacerbation of neuroinflammatory processes, leading to neuronal death and cognitive decline.^5^ Despite significant advances in our knowledge regarding tau neurobiology and the potential of targeting tau to deter neurodegenerative processes, few advances have been made in generating disease‐modifying therapies that modulate tau‐related pathology, at least in part due to limitations in commonly used animal models. In that light, nonhuman primates (NHPs) are an attractive alternative, displaying high sequence homology of proteins involved in AD pathophysiology and greater neuroanatomical transposability compared to humans.^6^, ^7^, ^8^

We have recently shown that injections of adeno‐associated virus capsid 1 (AAV1) carrying human 0N4R tau with two point mutations that occur individually in humans (AAV‐2xTau) into the entorhinal cortex (ERC) of adult rhesus macaques led to tau pathology spanning the ERC connectome, including the hippocampal formation (HF), compared to contralateral injections of a similar viral vector carrying green fluorescent protein (GFP).^9^ In the present study, we have expanded on those initial results and sought new insights into critical events underlying tau pathology by evaluating the spatiotemporal progression of tau in a similar NHP model. Toward that end, eight adult female rhesus macaques (10–16 years old) were randomly selected to receive stereotaxic injections of an AAV carrying an aggregation‐prone mutant of the human MAPT gene into the left ERC, and four additional age‐matched animals were used as controls, receiving injections of an empty‐vector control. Animals were subsequently divided into two cohorts with 3‐ and 6‐month endpoints when brains were collected and processed for three‐dimensional (3D) high‐resolution microscopic analyses. In addition to brain pathology at the two endpoints, baseline imaging scans (magnetic resonance imaging [MRI] and tau positron emission tomography [PET]) were performed prior to AAV administration, with a follow‐up scan performed at either 3 or 6 months. A panel of fluid biomarkers (cerebrospinal fluid [CSF] and plasma) was performed every 30 days, providing a comprehensive battery of noninvasive markers of neurodegeneration and neuroinflammation in a novel tau monkey model that recapitulates key features of tau pathology propagation in AD.

## 2 METHODS

### 2.1 Animals and neurosurgery

Twelve adult female rhesus macaques (10–16 years old – cycling, premenopausal) were selected among the animals in the California National Primate Research Center (CNPRC) animal colony to be employed in this study. Before the study, animals were prescreened for veterinary issues that could interfere with the interpretation of the results and then randomly assigned to each experimental group. Eight animals received unilateral stereotaxic intracortical injections of an adeno‐associated virus expressing a double‐mutated human tau‐targeting the left ERC (AAV1‐P301L/S320F, 1.176 × 10^13^ genomic copies/mL), while four control animals received unilateral injections in the left ERC of an empty vector (AAV1‐CTR) similar to the experimental group but lacking the tau payload. Surgeries were performed using the StealthStation surgical navigation system (Medtronic, Minneapolis, MN) using presurgery MRIs acquired for each animal for guidance. Two adjacent 18 μL injections encompassing all layers were made into the ERC, including layers II/III, for both AAV‐2xTau‐ and AAV‐CTR‐treated animals. Following injections, animals were randomly assigned to euthanasia at 3 (3 M group: AAV‐2xTau *n* = 4 and AAV‐CTR *n* = 2) or 6 (6 M group: AAV‐2xTau *n* = 4 and AAV‐CTR *n* = 2) months after surgery. Biofluid samples were longitudinally collected from all animals every 30 days after the injections, and animals received terminal MRI and PET scans before euthanasia. All experiments were conducted with approval by the Institutional Animal Care and Use Committee (IACUC) at the University of California–Davis (protocol no. 20752).

### 2.2 AAV preparation

The adenovirus employed in this work has been described in detail elsewhere.^9^, ^10^ Succinctly, a capsid 1 adenovirus was packaged with one copy of human 0N/4R tau‐containing two mutations (P301L/S320F) that render it more aggregation‐prone than wild‐type tau. The expression of this tau insertion is under the control of the hybrid cytomegalovirus enhancer/chicken β‐actin (CMV/CBA) promoter, a CBA intron (first intron of chicken β‐actin gene plus the splice acceptor of the rabbit β‐globin gene), woodchuck hepatitis virus post‐transcriptional regulatory element (WPRE), and bovine polyA. Virus packaging and purification were performed at the Penn Vector Core (University of Pennsylvania), following the golden standard of laboratory practices, including endotoxin testing (<5 endotoxin units per mL), purity testing (sodium dodecyl sulfate‐polyacrylamide gel electrophoresis), and titration (three rounds of digital polymerase chain reaction). After preparation, aliquots of AAVs were stored at −80°C until the surgeries.

**RESEARCH IN CONTEXT**

1. **Systematic review**: Using PubMed, the authors searched for nonhuman primate models of Alzheimer's disease (AD) that are more translatable to humans. Despite the presence of amyloid pathology being well‐documented in the literature as a common occurrence in monkeys during the aging process, these nonhuman primates generally do not develop tau pathology equivalent to humans.
2. **Interpretation**: Our results indicate that the delivery of the dual tau mutation in the entorhinal cortex generates tau‐based neuropathology and profound neuroinflammation in treated rhesus macaques, similar to what is observed in AD patients.
3. **Future directions**: By employing a combination of several approaches that are highly translatable to humans, our monkey model of AD can impact preclinical AD research and, in particular, further our knowledge of the earliest events reflecting tau‐based neurodegeneration and neuroinflammation, addressing the urgent need for alternative models of neurodegeneration.

### 2.3 CSF, serum, and plasma collection

Collections of CSF, plasma, and serum were performed prior to the surgery, once per month postsurgery, and immediately prior to euthanasia, totaling four collections for 3 M animals and seven collections for 6 M animals. For the collection of CSF, animals were sedated with ketamine (5–30 mg/kg, intramuscular) and dexmedetomidine (0.0075–0.015 mg/kg, intramuscular), a 23‐gauge spinal needle was inserted into the subarachnoid space of the cisterna magna, and 1–2 mL of CSF was aspirated, transferred to cryotubes, and stored at –80°C. For serum and plasma collection, ≈30 mL of venous blood was collected in EDTA‐containing tubes (plasma only) and centrifuged at 1500×*g* for 15 min. The samples were divided into 0.5 mL aliquots and stored at −80°C.

### 2.4 Perfusion and tissue preparation

Necropsy was performed by a team of trained pathologists in a biosafety level 2 environment. On the day of euthanasia, monkeys were deeply anesthetized with ketamine (25 mg/kg) and transcardially perfused with saline until the vascular bed was cleared, as described previously.^9^, ^11^ After perfusion, brains were harvested, hemisected, and sectioned into 6 mm‐thick coronal blocks. Blocks were fixed by immersion in 4% paraformaldehyde with 0.125% glutaraldehyde in phosphate buffer pH 7.4 at 4°C for 48 h under agitation. After the washes, blocks were stored in phosphate‐buffered saline (PBS) with 0.1% sodium azide at 4°C until processed. Semiseriated, 50 μm‐thick coronal sections were obtained by cutting blocks containing the ERC and HF in a vibratome. Sections were stored in PBS with 0.1% sodium azide at 4°C.

### 2.5 DAB (3,3′‐diaminobenzidine) immunohistochemistry (IHC)

An immunoperoxidase labeling method was used to visualize neurons with NEUN antibody (MAB377, Clone A60; MilliporeSigma, Burlington, MA). Endogenous peroxidase was quenched by 20‐minute incubation in 0.1 M sodium periodate, and background staining was blocked by 1‐hour incubation in a solution containing 2% bovine serum albumin and 5% normal horse serum. Tissue sections were incubated in PBS with NEUN primary antibody (1:1000) at room temperature overnight. After six washes, sections were sequentially incubated for 1 hour in biotinylated horse antimouse IgG (1:200; Vector, Burlingame, CA) followed by the *Elite* avidin‐biotin complex (1:500; Vector) for 75 minutes. The immunohistochemical reaction was completed with 0.05% DAB and 0.005% H_2_O_2_. Sections were mounted on gelatin‐coated slides, dehydrated through graded alcohol, cleared in xylene, and coverslipped with Cytoseal (Richard‐Allan Scientific, Kalamazoo, MI).

### 2.6 Stereological evaluation of number of NEUN‐immunoreactive neurons

An optical fractionator unbiased sampling design was used to estimate the number of NEUN‐immunoreactive neurons in CA1‐CA4, subiculum (SUB), and ERC cortex throughout their full rostrocaudal extent.^12^, ^13^, ^14^ The boundaries of ERC were determined according to the location of large, stellate, multipolar neurons in layer II and anatomic landmarks described from a monkey atlas.^15^ On the coronal sections, the medial border of ERC was limited rostrally by a group of dense neurons of the amygdala transition area and caudally by two groups of small neurons that belong to parasubiculum and presubiculum. The lateral border of ERC was restricted by rhinal fissure. On NEUN‐labeled sections, the entorhinal region, which is a six‐layered cortex, was outlined under low magnification (1.25X) and was defined consistently at all levels of each series of sections in each case.

The SUB lies between the ERC and the CA1 subfield of the hippocampus proper and shows a gradual transition from a six‐ to a three‐layered cellular organization. The border between parasubiculum and ERC was described above. The border between prosubiculum and CA1 was limited according to neuronal morphology. The neurons in prosubiculum are of higher density and smaller sizes. All subfields of the subiculum were outlined as a whole for stereological evaluation.

The CA1 and CA2 (CA1/2) were outlined as a unit because CA2 is a small region. CA1/2 has three fundamental layers and the characteristics in CA1/2 are NEUN‐labeled large pyramidal cells with apical dendrites. The CA3 region is located near to the dentate gyrus (DG) with a light staining region which contains Mossy fibers and CA4 underlies hilar region. As there is no obvious border between CA3 and CA4 region, the CA3/4 was used as a unit for stereological evaluation. Approximately three to five equispaced sections were sampled from each brain area. The section sampling fraction (SSF) was 1/0.042. The distance between sections was approximately 0.96 mm.

The ERC, SUB, CA1/2, and CA3/4 were outlined separately using a 1.25× objective. A systematic sample of the area was made from a random starting point (StereoInvestigator v10.40 software; Micro‐BrightField, Colchester, VT). Counts were made at regular predetermined intervals (x = 313 μm, y = 313 μm), and a counting frame (70 × 70 μm = 4900 μm^2^) was superimposed on images obtained from tissue sections. The area sampling fraction (asf) was 1/0.05. These sections were then analyzed using a 60× Planapo oil immersion objective with a 1.4 numerical aperture. The section's thickness was empirically determined. Briefly, as the top of the section was first brought into focus, the stage was zeroed at the z‐axis by software. The stage then stepped through the z‐axis until the bottom of the section was in focus. Section thickness averaged 20.21 ± 2.3 μm. The disector height (counting frame thickness) was 10 μm. This method allowed for 1 μm top guard zones and at least 2 μm bottom guard zones. The thickness sampling fraction (tsf) was 1/0.51. Care was taken to ensure that the top and bottom forbidden planes were never included in the cell counting. Using stereological principles, NEUN positive neurons in each case were sampled using a uniform, systematic, and random design. The number of NEUN neurons within ERC, SUB, CA1/2 and CA3/4 was calculated separately using the following formula: *N* = ΣQ^−^·1/ssf · 1/asf · 1/tsf. ΣQ^−^ was the number of raw counts.

The volume of ERC, SUB, CA1/2, and CA3/4 regions was estimated using Cavalieri estimator (StereoInvestigator v10.40 software).^16^ The densities of NEUN positive neurons were separately calibrated by estimated neuronal number from optical fractionator/volume from Cavalieri estimator (neuronal number/mm^3^). The coefficients of error (CE) were calculated according to the procedure of Gunderson and colleagues as estimates of precision.^16^, ^17^ The values of CE were 0.10 ± 0.02 (range 0.08–0.12) in ERC and SUB and 0.12 ± 0.05 (range 0.10–0.15) in CA1/2 and CA3/4 regions.

### 2.7 Immunofluorescence and histological staining

Immunofluorescence was employed in this study for the biochemical characterization and neuroimmunological experiments, based on the protocol described in Beckman et al.^11^ Briefly, antigen retrieval was performed by incubating free‐floating brain sections with citrate buffer pH 6.0 (S1700; FUJIFILM Wako, Osaka, Japan) at 60°C for 30 min. After washes in PBS, off‐target antibody binding blocking and membrane permeabilization were performed by incubating sections with a 5% normal donkey serum, 5% normal goat serum, and 5% bovine serum albumin solution in PBS with 0.3% triton for 2 h at room temperature under agitation. Sections were then incubated at 4°C for 48 h with up to four of the following antibodies:

- Neuronal markers: NEUN (1:1000; 266 004, Synaptic Systems GmbH, Göttingen, Germany), MAP2 (microtubule‐associated protein 2; 1:1000; 188 004, Synaptic Systems), PSD95 (postsynaptic density protein 95; 1:1000; ab12093, Abcam, Cambridge, UK), Pan‐Neurofilament (1:400; DMAB 7133, Creative Diagnostics, New York, NY), Parvalbumin (1:500; 195 004, Synaptic Systems).
- Tau epitopes: AT8 (1:500; MN1020, Invitrogen, Waltham, MA), Tau 3R (1:400; 2A1‐1F4, FUJIFILM Wako), Tau 4R (1:500; ab218314, Abcam), pS396 (1:1000; ab109390, Abcam), pS422 (1:1000; ab79415, Abcam). The following antibodies were all generously provided by Dr. Nicholas Kanaan: TOC1 (1:1000), TauC3 (1:1000), TNT2 (1:1000), Alz50 (1:1000), Tau5 (1:1000), PHF1 (1:1000), MN423 (1:1000).
- Neuroinflammation: IBA1 (ionized calcium‐binding adapter molecule 1; 1:500; 019‐19741, FUJIFILM Wako), HLA‐DR (human leukocyte antigen – DR complex; 1:200; MA5‐11966, Invitrogen), TREM2 (triggering receptor expressed on myeloid cells 2; 1:500; AF1828, R&D Systems, Minneapolis, MN), GFAP (glial fibrillary protein; 1:1000; ab4674, Abcam).

Following incubation with primary antibodies, sections were thoroughly washed with PBS and incubated with the appropriate fluorophore‐conjugated secondary antibodies (Alexa Fluor 405, 488, 555, and 647; Invitrogen) at 1:500 dilution for 2 h at room temperature or 48 h at 4°C, depending on the antigen. Finally, nuclei were counterstained with 4′,6‐diamidino‐2‐phenylindole (DAPI 0.001 mg/mL; Invitrogen), when applicable, followed by an autofluorescence eliminator reagent (MilliporeSigma) to minimize lipofuscin autofluorescence. Sections were placed onto glass microscopy slides, mounted with antifading mounting media (Prolong Diamond; Invitrogen), and coverslipped.

In addition to the IHC procedure described above, some sections were subject to Thioflavin S (ThioS) staining for the identification of mature neurofibrillary tangles (NFTs) and ghost tangles. Following DAPI counterstaining, free‐floating sections were briefly rinsed in PBS and then incubated in a 0.05% ThioS solution for 20 min. Sections were then rinsed in 80% ethanol for 3 minutes and PBS for 10 min and mounted as described above.

### 2.8 Microscopy and image analysis

All images were acquired in 3D (z‐stacks ranging from 25 to 50 μm) using an upright microscope AxioImager Z2 (Carl Zeiss AG, Oberkochen, Germany) equipped with an LSM800 confocal head and a 32‐channel Airyscan detector (Zeiss). Acquired images were processed using the semiautomated 3D segmentation software Imaris 9.8 (Bitplane, Belfast, UK), as described below:

- NEUN/AT8/ThioS colocalization—Three 20× images were acquired from each hippocampal region analyzed in this study (CA3, CA1, SUB, left ERC [L‐ERC], and right ERC [R‐ERC]), with at least 240 NEUN+ cells analyzed per region, per animal. Each marker was individually segmented, surfaces were rendered in 3D, and objects were counted to produce total counts per mm^3^.
- AT8/3R and AT8/4R colocalization—Three 20× images were acquired from the CA3/hilus and layer II of the ERC. Each marker was individually segmented, surfaces were rendered in 3D, and objects were counted to produce total numbers. The total number of 3R and 4R objects colocalized with AT8 was then divided by the total number of AT8+ cells and reported as the percentual of colocalization.
- Microglia morphology and PSD95 engulfment – For each animal, three 50 μm sections of medial HF + ERC were chosen for imaging. Four to eight fields were imaged in the ipsilateral territory (left side, AAV‐injected) and four to eight fields were imaged in the contralateral territory (right side, noninjected), with a minimum of eight fields per HF, 16 slides, per animal. A 3D volume surface rendering of each z‐stack was created to determine the volume of the microglia, synaptic markers, and tau inputs. To visualize and measure the volume of engulfed material, any fluorescence that was not within the microglia and/or lysosome volume was subtracted from the image. The remaining engulfed material was surface rendered using parameters previously determined for all synaptic inputs and the total volume of engulfed inputs was calculated.^9^, ^11^ To determine % engulfment, the following calculation was used: Volume of internalized synaptic input (μm^3^)/Total volume microglial cell (μm^3^).
- 3D Colocalization of tau markers—For each animal, 12 50 μm sections covering ERC and HF regions were chosen for imaging and analysis. Three to six fields were imaged at 20×, in the ipsilateral territory (left side, AAV‐injected), and four to eight fields were imaged in the contralateral territory (right side, noninjected), with a minimum of eight fields per region, 16 slides, per animal. The regions analyzed cover the CA1/2, CA3/4, SUB, and layer 2 of ERC.

### 2.9 MRI

Structural imaging of the monkey brains was performed using a 3T MRI scanner (Skyra, Siemens Healthcare, Germany) with an 8‐channel receiver coil optimized for monkey brain scanning (Rapid MRI, Columbus, OH). T_1_‐weighted images were acquired using the magnetization‐prepared rapid acquisition with gradient echo (MPRAGE) pulse sequence with the following parameters: field of view = 154×154 mm; 480 sagittal slices; TR/TE = 2500/3.65 ms; flip angle = 7°; TI = 1100 ms; voxel size 0.6×0.6×0.6 mm, interpolated to a resolution of 0.3×0.3×0.3 mm.

The T_1_‐weighted MRI scans were used for brain segmentation. Segmentation of the hippocampus was performed using DARTEL SPM12 (Welcome Trust Centre for Neuroimaging, London) in MATLAB (The MathWorks, Inc; Natick, MA). A pipeline incorporating all the preprocessing steps, including bias field correction, skull stripping, coregistration between time‐points, tissue segmentation, normalization, and reverse transformation, was applied to 12 animals. First, MR images were segmented into gray matter (GM), white matter (WM), and CSF by matching each tissue type to the corresponding tissue in the standard space of the Montreal Neurological Institute (MNI) Rhesus Macaque Atlas.^18^ In the next step, a GM template specific to our datasets was generated through iterative registration. The reverse transformation from each subject to this GM template was applied to the atlas mask to acquire individual hippocampus masks in the native space.

### 2.10 PET radiotracer synthesis

APRINOIA Therapeutics (Taipei City, Taiwan) provided the precursor and a description of methods used for the radiosynthesis of [^18^F]APN1607.^19^ Briefly, cyclotron‐produced [^18^F]fluoride was delivered to the Tracerlab FX2 and loaded on a preconditioned QMA SPE (Waters 186004540). ^18^F was eluted to the reaction vessel in K.222 (7.1 mg) and K_2_CO_3_ (0.7 mg) in CH_3_CN (1.2 mL) and H_2_O (0.3 mL). The ^18^F was dried (110°C), dissolved in CH_3_CN (1.5 mL), and dried again (100°C). The precursor (2.5 mg) in dimethyl sulfoxide (DMSO) (1.5 mL) was added, the reaction vessel was pressurized to 225 kPa, and the fluorination reaction was performed at 110°C for 10 minutes. Then, 3 M HCl (0.8 mL) was added, the reaction vessel was pressurized, and hydrolysis was performed at 90°C for 4 minutes. The reaction solution was cooled, 0.25 M NaOH (10 mL) was added, and the crude product was loaded on a preconditioned tC18 cartridge (Waters WAT036810). The cartridge was washed in H_2_O (5 mL), then eluted in EtOH (2 mL). The crude product in EtOH was added to 5 mg/mL sodium ascorbate in H_2_O (3 mL) and transferred to an HPLC loading loop. Purification HPLC was performed with a Luna C18 10 mm × 250 mm column eluted in 60% EtOH/40%—5 mg/mL sodium ascorbate (aqueous) at 2.8 mL/min. Purified [^18^F]APN1607 was recovered with a retention time of approximately 10 minutes. [^18^F]APN1607 was formulated in 5 mg/mL sodium ascorbate such that the concentration of EtOH was adjusted to 10% by volume, and transferred with syringe end filtration (0.22 μm) into a sterile amber vial. The entire process from radiosynthesis to injection of [^18^F]APN1607 was performed under UV‐cut light to avoid photoisomerization. Over the 12 production runs, the radiochemical purity was >97% at the end of synthesis, and molar activity at radiotracer injection was 189.39 [156.70, 297.27] GBq/μmol (median [95% confidence interval {CI}]).

### 2.11 PET imaging

Scans were conducted using a dedicated monkey brain PET scanner (PiPET, Brain Biosciences, Rockville, MD). Monkeys were positioned head‐first supine with the brain centered in the scanner's field of view. After a 10‐minute cold transmission scan, [^18^F]APN1607 was injected at a dose of 149.85 ± 11.84 MBq. A 120‐min dynamic emission PET scan was initiated starting approximately 10 s prior to injection of [^18^F]APN1607. Data were reconstructed using the maximum likelihood expectation maximization method into 27 frames based on the following framing: 4×15s, 4×60s, 5×180s, 6×300s, and 6×600s.

All data analysis was performed using PMOD version 4.002. Averaged PET images using frames 23‐26 were generated from the dynamic data, with the PET images co‐registered to an individual MRI which was normalized to a template NHP MRI (INIA19). The co‐registered PET images were then also normalized using the same transformation parameters. Regions of interest (ROIs) were delineated on the template MRI and included the ERC, fusiform, precuneus, posterior cingulate cortex, and posterior parahippocampal gyrus. Standardized uptake value ratio (SUVR) images were generated by using the cerebellum as a reference, with the overall change in SUVR from baseline (DSUVR) at 3 or 6 months post‐AAV obtained by subtracting the SUVR images obtained prior to AAV administration (PET1) from the SUVR images 3 or 6 months post‐AAV administration (PET2). A positive DSUVR value indicates an increase in SUVR following AAV administration.

### 2.12 Fluid biomarkers

Validation of human enzyme‐linked immunosorbent assays (ELISAs) using rhesus monkey samples was performed in a previous study.^11^ Assays for total tau (t‐tau), p‐tau Thr181, p‐tau Ser199, p‐tau Thr231, p‐tau Ser396 (Invitrogen), tumor necrosis factor alpha (TNF‐α), interleukin 6 (IL‐6), TREM2, brain‐derived neurotrophic factor (BDNF) (Abcam), and neurofilament‐light (NF‐L; Uman Diagnostics AB, Umeå, Sweden) were performed according to each kit manufacturer's instructions, after sample dilution optimization. Following ELISA, the optical density of each sample was measured using a spectrophotometer and compared to a standard curve to determine the nominal content of each analyte. Samples were run in duplicate, and the results reported in this study correspond to the average of the technical replicates.

### 2.13 Statistical analysis

All analyses were performed with GraphPad Prism 6, and datasets were assessed for normality parameters prior to significance determination. Values are expressed as means ± standard error of the mean (SEM). Statistical tests and p values are indicated in each figure legend.

## 3 RESULTS

### 3.1 AAV‐2xTau delivery in the ERC induces progressive tau pathology in rhesus monkeys

**FIGURE 1:**
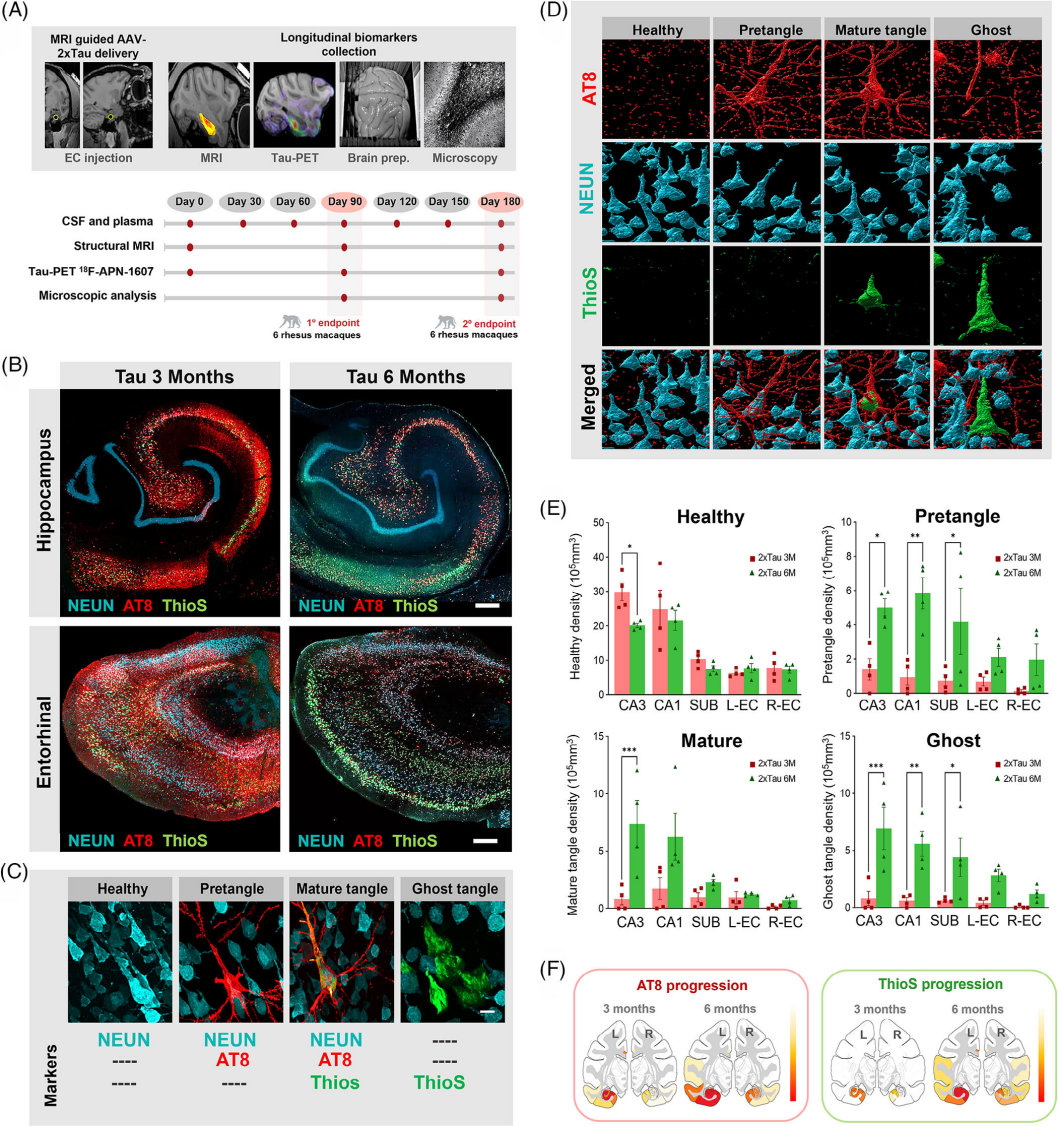
Quantitative 3D analysis of AT8 and ThioS pathology progression in AAV‐2xTau‐injected animals. (A) A summary of the experimental procedures and longitudinal sample collection performed in this study. (B) Fluorescence photomicrographs illustrating the distribution of NEUN (blue), AT8 (red), and ThioS (green) in the hippocampal formation and entorhinal cortex of experimental animals at 3 and 6 months. Tau pathology progression is highlighted by the increased ThioS staining observed in 6‐month animals compared to 3‐month animals. (C) Representative confocal images of the markers examined to quantify disease progression and the four typical neuronal profiles commonly observed in the analyzed regions: healthy, pretangle, mature tangle, and ghost tangle. (D) Three‐dimensional reconstruction of high‐resolution confocal images was performed to identify and quantify tau pathology progression. Expression of each neuronal profile: healthy, pretangle, mature, and ghost tangles, was calculated and corrected for the 3D volume (mm3) occupied by each region analyzed: CA3/hilus, CA1, subiculum (SUB), left ERC, and contralateral ERC (E). A Graphical summary showing the pattern of distribution of AT8 and ThioS across the left and right hemispheres and between the two‐time points investigated is shown in (F). Scale bar: 200 μm (b), 10 μm (c). *p < 0.05 **p < 0.01 ***p < 0.001, two‐way ANOVA, Sidak's post hoc test.

The methodology and experimental approach used in this study, including biomarker collection and endpoints, as well as detailed brain regions used for stereological analysis are summarized in Figure 1A and Supplementary Figure 1. To determine the spatiotemporal progression of misfolded tau following AAV‐2xTau delivery, the neuronal marker NEUN was combined with the phospho‐tau marker AT8 (p‐Tau S202/T205, often associated with paired‐helical formation), and ThioS (labels β‐sheet fibrils). In agreement with our previous report,^9^ exogenous tau expressions in the ERC resulted in significant AT8 phosphorylation in the 3 M group, while ThioS was comparatively more restricted. In the 6 M group, however, there was a shift in the proportion of AT8 and ThioS+ cells, with the latter becoming substantially more numerous (Figure 1B). We then performed stereological semiautomated 3D segmentation to classify neurons based on their stage of tangle formation (Figure 1C,D). In the CA3 region, our study revealed a 32% reduction in the healthy neuronal population (NEUN+/AT8‐/ThioS‐) in the 6 M group compared to 3 M animals (*p* = 0.0294). Analysis of the pretangle neuronal population (NEUN+/AT8+/ThioS‐) demonstrated a significant increase in this profile at 6 M compared to 3 M across all hippocampal regions analyzed: a three‐fold increase in CA3 (*p* = 0.0204), six‐fold in the CA1 (*p* = 0.0011), and five‐fold in the SUB (*p* = 0.0296). Investigation of the presence of mature tangles (NEUN+/AT8+/ThioS+) highlighted the later stage of tau pathology observed in the 6 M animals compared to 3 M: an increase of 9‐fold was observed in the CA3 (*p* = 0.0004), followed by an increase of 3.6‐fold in the CA1 and 2.3‐fold in the SUB (not statistically significant). Finally, analysis of the ghost tangle population (NEUN‐/AT8‐/ThioS+) suggested a time‐dependent increase in late‐stage pathology. When compared to the 3 M group, 6 M animals had an 8.8‐fold increase in the ghost tangle population in the CA3 (*p* = 0.0002), 9.3‐fold in the CA1 (*p* = 0.0027), and 7.2‐fold in the SUB (*p* = 0.03). No significant increases were observed in the treated and contralateral ERC region (Figure 1E). Outside the HF and ERC region, additional temporal spreading of tau pathology was also observed in connected cortical areas such as the retrosplenial cortex and inferior temporal gyrus (Figure 1F). Similar analysis was performed in animals from the control group injected with an AAV‐empty vector, but no misfolded tau or neurofibrillary tangles were detected in controls from the 3 and 6 M groups (Supplementary Figure 2). Taken together, these results confirm the nature of misfolded tau progressively spreading across interconnected areas following AAV‐2xTau injection, consistent with the tau pathology observed in AD patients.^20^

### 3.2 Exogenous tau‐induced pathology templates endogenous tau generating previously established biochemical intermediaries

**FIGURE 2:**
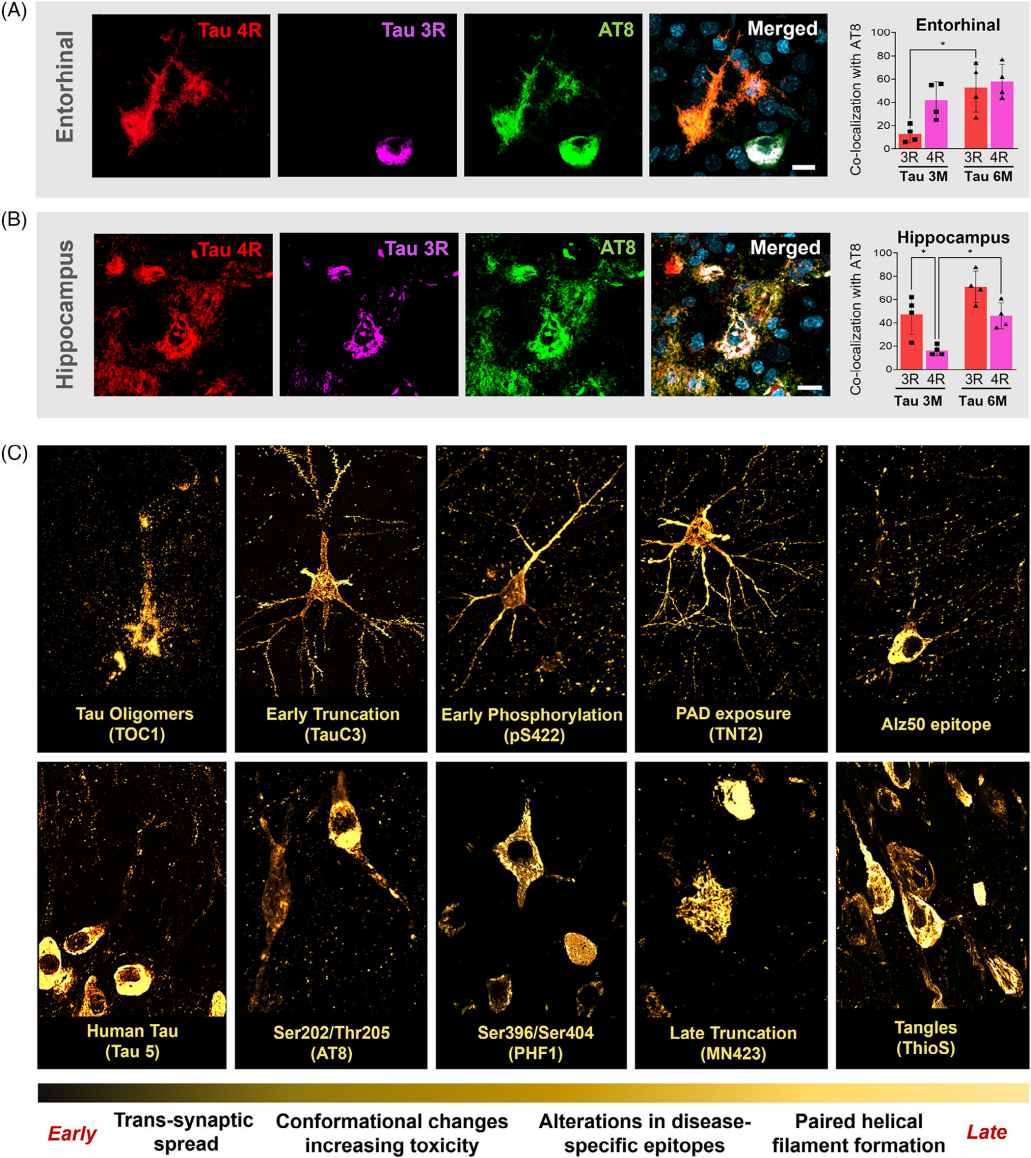
Comprehensive analysis of the biochemical progression of tau pathology in the AAV‐2×Tau monkey model. As abnormal splicing in both 3R and 4R tau isoforms is known to be involved in the development of tau pathology in AD, we investigated the expression of both isoforms and their colocalization with AT8. Quantification was performed in the ERC (A) and in the CA1 region of the HIP (B) after the AAV‐2×Tau injection. To further investigate the profile of tau‐induced pathology in treated monkeys, we analyzed a panel of 10 tau‐associated epitopes detected in the ERC‐HF area (C). The TOC1 antibody was used to target tau oligomers, often found in a punctate pattern over cell somas and proximal dendrites. The Alz50 antibody labels early tau phosphorylation, generating diffuse labeling over the soma and abundant labeling of apical and basal dendrites. The antibody specific for phosphorylated tau at S422 produces abundant labeling of somas, dendrites, and axons. TNT2 marker, which identifies an epitope comprised of amino acids 7‐12 present in AD brains, produced highly specific labeling, including a very strong immunosignal in the soma, dendrites, and dendritic spines (highlighted). Antibody TauC3, which labels an early truncated form of tau, produced strong somatic and basal dendritic labeling. The Tau5 antibody that does not bind to rhesus tau, identified human tau protein originated from the viral vector expression, as opposed to endogenous templated tau. The transition from early and intermediate stages of tau alterations into late tangle‐forming species is characterized by predominant somatic staining with a relatively lower contribution of the dendritic compartment, as visualized through antibodies for phospho‐epitopes PHF1 and AT8. The labeling pattern obtained with antibody MN423, a marker for late tau truncation, reveals neurons with substantial morphological alterations and numerous damaged neurites. Finally, ThioS staining was successful in revealing tangles and extracellular remains of tangle‐bearing neurons. Scale bar: 10 μm, *p < 0.05, two‐way ANOVA, Tukey's post hoc test.

Considering the expansion of tau pathology between 3 and 6 months, we hypothesized that the exogenous tau might permissively template endogenous tau into aggregation‐prone forms. To test this hypothesis, we leveraged the fact that the viral construct employed carries a 0N/4R tau isoform solely, allowing us to use the colocalization between 3R/phosphorylated tau as a proxy for the recruitment of endogenous tau into pathology‐associated forms. In the ipsilateral layer II of ERC, there is a preponderance of the AT8/4R+ population (42%) when compared to the AT8/3R+ neuronal population (12.75%) at the 3‐month time‐point. In the 6‐month group, there is an increase of approximately 40% in the total number of AT8/3R neurons (from 12.75% to 53%, *p* = 0.0187), while the AT8/4R population increased by only 16% (from 42% to 58%; Figure 2A). In the CA1 region of the HF, conversely, AT8/3R co‐expression was observed in 47.25% of the neurons in the 3 M group and increased to 71% in the 6 M group. AT8/4R colocalization was detected only in 16.25% of the neurons of the CA1 region, increasing 2.8 times in the 6‐month group (46%, Figure 2B, *p* = 0.0283). These results suggest that, while the viral expression of 2xTau is responsible for quickly generating a population of AT8/4R expressing neurons at the injection site at 3 months, both the expansion of that population and the spread of tau pathology to other brain areas are driven primarily by the templating of endogenous tau into phosphorylation‐ and aggregation‐prone molecular intermediaries.

We then investigated if the pathological tau templating observed in the macaque produced previously described molecular intermediaries, particularly early modifications highly associated with the toxic actions of tau^21^, ^22^, ^23^, ^24^, ^25^, ^26^, ^27^ (Figure 2C). Early pathological events, such as the formation of tau oligomers (TOC1),^21^, ^25^ could be detected across the temporal lobe of both 3 and 6 M animals, but not in CTR animals. Other conformational changes linked to increased tau toxicity, such as the misfolded isoform recognized by Alz50^24^, ^26^ and tau phosphorylation at S422,^23^ were predominantly detected in the HF‐ERC region of treated macaques. Notably, an epitope linked to the early exposure of a tau active motif in the C terminus associated with the start of axonal transport impairment (TNT2)^26^, ^27^ was found in a diffuse neuronal labeling pattern, including dendrites and spines. Furthermore, we were able to identify a truncated form of tau generated by the caspase‐mediated cleavage of tau at D421 (TauC3),^23^ as well as immunoreactivity associated with the Tau5 antibody—which selectively does not bind to rhesus tau^24^—reflecting the presence of human tau derived from the viral expression across the hippocampal region. Pathological phospho‐epitopes, such as PHF1 (phospho‐tau S396/S404) and AT8, were found most concentrated in the soma and dendrites, consistent with the hypothesis that hyperphosphorylated tau is redistributed from the axon to other cellular compartments due to microtubule destabilization.^28^ In the hippocampus of 6 M macaques, we identified the presence of late tau truncation at glutamic acid^391^ (MN423)^29^ often in the absence of NEUN or MAP2 colocalization, suggesting advanced tangle formation and severe compromise of neuronal function. Finally, we observed ghost tangles lacking an intact neuronal membrane by directly staining beta‐pleated sheets using ThioS (Figure 2C).

### 3.3 Tau spread in the AAV‐2×Tau model is reflected in CSF biomarkers and in vivo imaging

**FIGURE 3:**
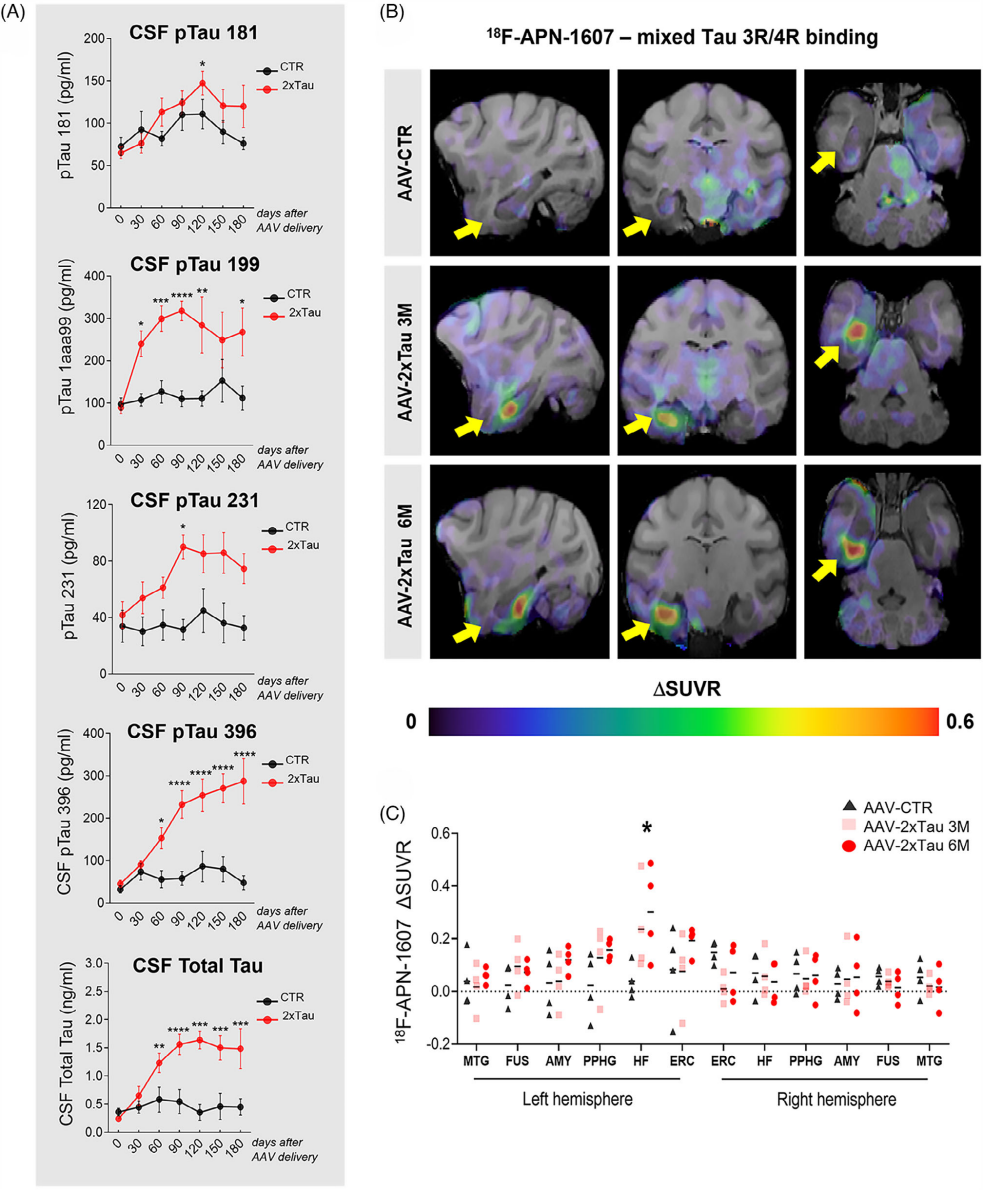
Progressive hippocampal atrophy positively correlates with AD‐related fluid biomarkers following AAV‐2×Tau injection. Analysis of tau pathology progression in the CSF was performed in all experimental groups. Different phosphorylated tau epitopes such as threonine 181, serine 199, threonine 231, and serine 396, as well as total tau, were longitudinally analyzed following AAV genetic delivery, reflecting tau pathology accumulation in the CSF as a proxy for AD‐related brain pathology (A) [^18^F]APN‐1607, was used to visualize misfolded tau progression after AAV delivery across the different experimental groups (B). Changes in standardized uptake value ratio (DSUVR) for [^18^F]APN‐1607 in selected brain regions across both hemispheres are shown in (C). ERC = entorhinal cortex, HF = hippocampus, PPHG = posterior parahippocampal gyrus, AMY = amygdala, FUS = fusiform gyrus, MTG = middle temporal gyrus. *p < 0.05 **p < 0.01, ***p < 0.001, ****p < 0.0001, two‐way ANOVA, Tukey's post hoc test.

After establishing the initiation and spread of tau pathology following intracortical injections of AAV‐2×Tau, we investigated both CSF biomarkers and in vivo imaging to assess the extent to which clinically relevant tools can reflect tauopathy progression in our NHP model. Toward that goal, we first evaluated the longitudinal concentration profile in the CSF of four different phosphorylated tau epitopes and total tau, known to be increased in AD patients^30^ (Figure 3A). Tau phosphorylated at threonine 181 remained close to the baseline throughout the experiment, becoming significantly higher only on day 120 (*p* = 0.0247). On the other hand, tau phosphorylated at serine 199 was the most rapidly changing tau biomarker identified in this study, becoming significantly different from the baseline as soon as 30 days after injection and remaining significantly elevated until day 120 and then again on day 180 (*p* = 0.0259). Tau phosphorylated at threonine 231 reached statistical significance only at 90 days (*p* = 0.0152). Tau phosphorylated at serine 396 significantly increased from day 60 (*p* = 0.0418) through day 180, when necropsy occurred. Finally, there was a substantial increase in the total tau concentration in the CSF of experimental animals, reaching statistical significance at 60 days after viral delivery (*p* = 0.0012) and remaining high for the duration of the experiment (180 days) after reaching a plateau at around 120 days.

Second, taking advantage of the recently developed tau PET tracer [^18^F]APN‐1607 (formerly known as [^18^F]‐PM‐PBB3), which appears to bind to both AD and non‐AD tau,^31^ we investigated in vivo correlates of the tau pathology observed in our histopathological examinations. AAV‐2×Tau delivery resulted in increased [^18^F]APN‐1607 uptake across the left ERC and HF at both time points when compared to the empty vector delivery, indicating that the tau overexpression caused by the AAV administration can be detected via in vivo PET imaging with [^18^F]APN‐1607 (Figure 3B). The change in SUVR from baseline to 3‐ or 6‐month post‐AAV administration (DSUVR) for [^18^F]APN‐1607 revealed a statistically significant group effect in the left hippocampus (*p* = 0.0448), with the 6M groups displaying a significantly higher shift from background (DSUVR = 0.30 ± 0.09 [SEM]) compared to empty vector‐injected animals (Figure 3C). Considering that [^18^F]APN‐1607 is a highly specific tau tracer,^31^ its detection in an NHP model using a noninvasive technique reinforces the translational power of the AAV‐2×Tau monkey to investigate future interventions for AD.

### 3.4 Virally induced tau pathology is associated with neurodegeneration in the ERC and HF

**FIGURE 4:**
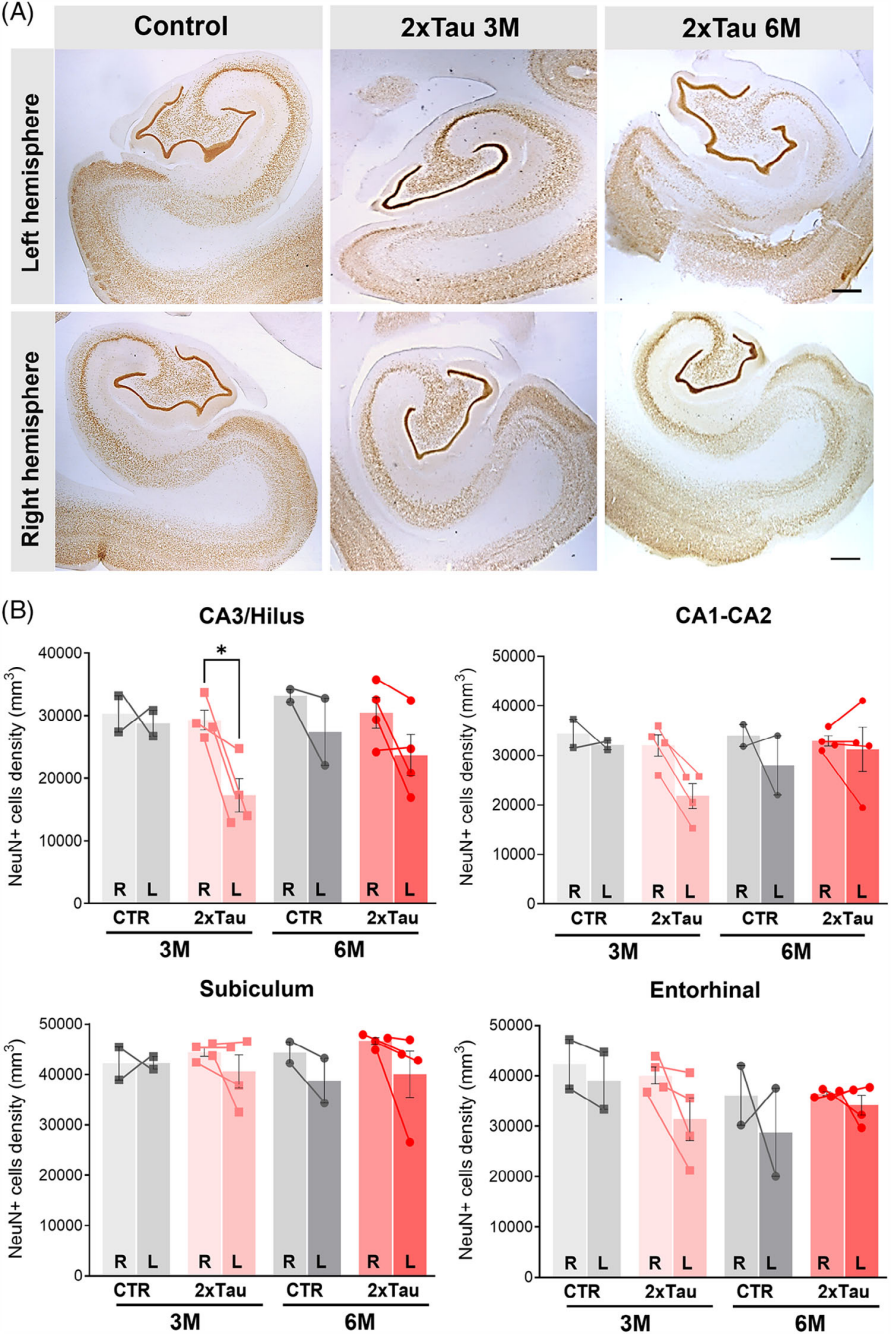
Unbiased stereological counting demonstrates selective neuronal loss across hippocampal regions following ERC injection. (A) Stereological NEUN counting was performed using DAB chromogenic staining across the left (injected side) and the right (contralateral) hemispheres. (B) Total NEUN+ cell density was calculated for the CA3/hilus region, CA1‐CA2, subiculum (SUB), and left entorhinal (L‐ERC). Scale bar: 20 μm. *p < 0.05 **p < 0.01, two‐way ANOVA, Sidak's post hoc test.

To investigate if the tau pathology observed in this model is associated with neuronal loss, we performed stereological counts of NEUN+ cells in the ERC and HF of both hemispheres using DAB across all experimental groups (Figure 4A,B). In the left CA3/Hilus, the total number of neurons significantly decreased by 35% comparing 3 and 6 M groups (*p* = 0.0165). No statistically significant differences were observed in CA1/CA2 or SUB, despite a nominal reduction in the number of NEUN+ cells in the left hemisphere compared to the right hemisphere at both 3 and 6 months postdelivery. Finally, in the left ERC, no statistically significant differences were observed (Figure 4B). These results suggest a selective progressive neuronal loss occurring in the CA3/hilus region first. In addition to frank neuronal loss, we also observed a degradation of the neuronal cytoskeleton, as revealed by a loss of pan‐Neurofilament when tau pS396 is present inside neurons (Supplementary Figure 3), suggesting the tau‐mediated neuronal damage and dysfunction also happen in the absence of complete neuronal loss.

**FIGURE 5:**
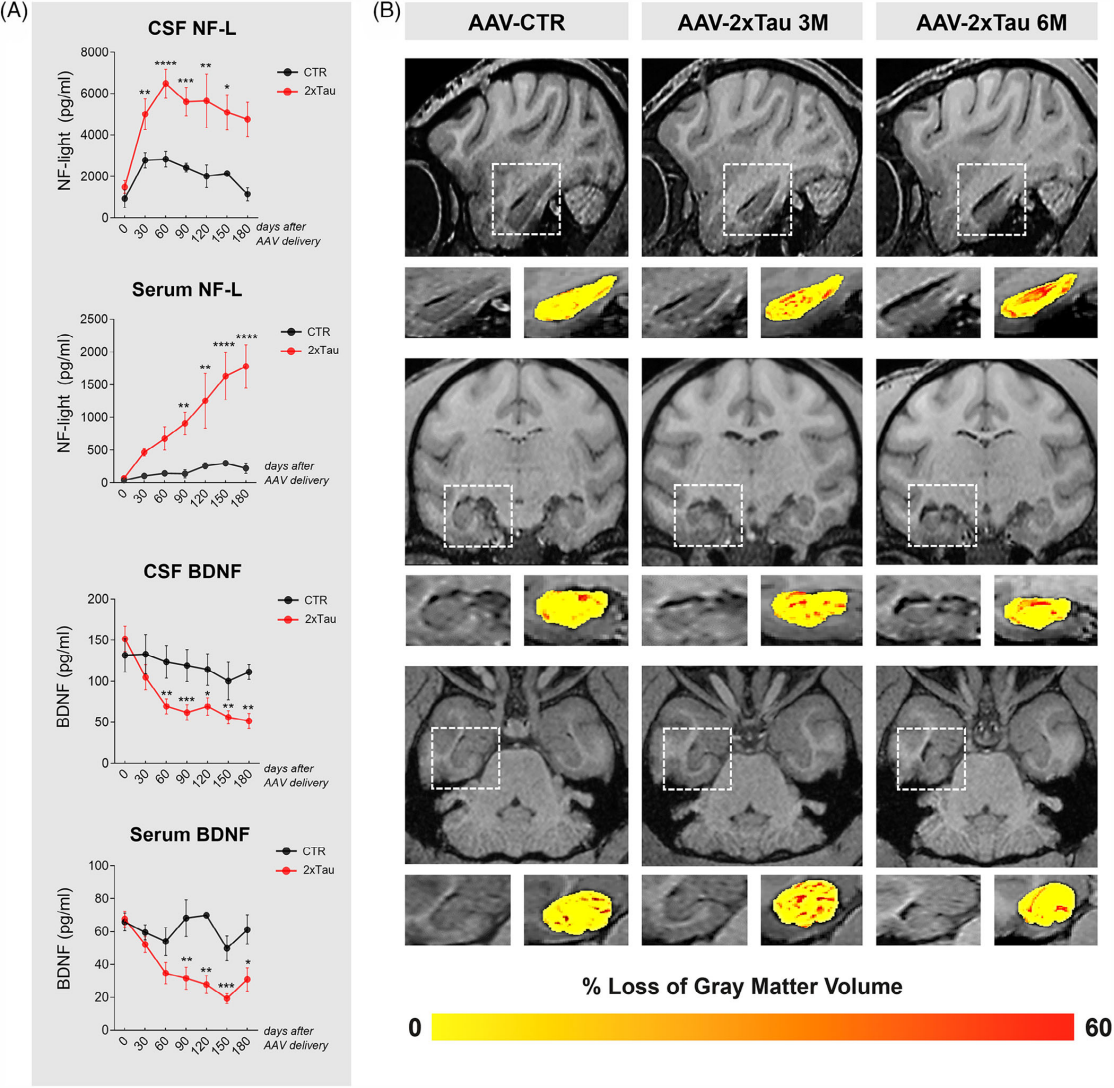
Progressive hippocampal atrophy positively correlates with AD‐related fluid biomarkers following AAV‐2×Tau injection in rhesus macaques. Longitudinal representation of neurodegeneration‐related biomarkers in the CSF and plasma of control and experimental animals. A robust increase in NF‐L levels in the CSF and plasma was detected early in the disease progression, and levels stayed elevated in the following months until the endpoint collection. (A) Notably, a robust reduction in BDNF levels was also detected early in disease development in both the CSF and plasma. MRI scans were performed in all experimental groups prior to AAV delivery and at the 3‐ and 6‐month time‐points. (B) Representative images for each animal group. These images were collectively used to calculate overall loss of gray matter volume in the hippocampus relative to the baseline. The degree of severity was visually color‐coded by yellow to orange to indicate increasing atrophy, within the white‐squared selected zoom images. (C) Longitudinal volumetric analysis of the hippocampus was calculated for each experimental group, and the correlation of the MRI volumetric changes and NEUN density in the hippocampus, as well as with endpoint NF‐light CSF levels. *p < 0.05 **p < 0.01, ***p < 0.001, ****p < 0.0001, two‐way ANOVA, Tukey's post hoc test.

An additional investigation for core biomarkers of neurodegeneration was performed using CSF and plasma collected monthly from treated monkeys (Figure 5A). Neurofilament‐light is a putative biomarker in several neurodegenerative conditions and has been shown to correlate with brain atrophy and cognition in AD patients.^32^ We detected a robust increase in NF‐L levels in the CSF and plasma of AAV‐2×Tau‐injected animals. An increase in CSF NF‐L was also observed initially in CTR animals, possibly caused by the neurosurgery for AAV injections, as the levels reduced with time in the following monthly collections. In contrast, CSF NF‐L levels remained elevated in the 2×Tau treated animals compared to AAV‐CTR animals, and at 60 days after CSF collection, the levels were around 2.3x higher in 2×Tau compared to the CTR group (CTR: 2835 vs. 2×Tau: 6480 pg/mL, *p* = 0.031). In the plasma, NF‐L levels were consistent across the CTR animals but progressively increased with time in 2×Tau animals, especially 90‐180 days after injections (CTR: 301 vs. 2×Tau: 1632 pg/mL, *p* = 0.0252 – 150 days; CTR: 225 vs. 2×Tau: 1780 pg/mL, *p* = 0.0038 – 180 days). Brain‐derived neurotrophic factor is a neuroprotective factor with a pivotal role in neuronal survival and is known to be downregulated by tau pathology in AD patients, as well as cellular and animal models of AD.^33^ Investigation of BDNF levels in treated monkeys showed a sharp decrease in both the CSF and plasma of 2×Tau animals. In the CSF, BDNF levels were consistently lower by 45%‐60% across all time‐points in 2×Tau animals compared to CTR, while plasma levels were also reduced by half in 2×Tau compared to CTR and were notably different at the 90‐day time‐point (CTR: 68.17 vs. 2×Tau: 31.64 pg/mL, *p* = 0.0143).

To complement the histological analyses, MRI scans were performed in all groups prior to AAV delivery and the 3‐ and 6‐month time‐points. Illustrative cross‐sectional views of the MRI datasets with the hippocampal volume highlighted are presented in Figure 5B. Volumetric analysis of the hippocampus for each experimental group revealed a progressive decrease in hippocampal volume between AAV‐2×Tau groups (−8.4% ± 2.4% [SEM] at 3 M, −14.6% ± 3.5% at 6 M) and controls (−3.5% ± 0.6%), suggesting the degree of neurodegeneration observed in this study can be detected by in vivo imaging. To understand how this trend correlates with other markers of neurodegeneration investigated in this study, we performed correlative analyses between the MRI volume change, the density of NEUN+ cells, and the content of NF‐L in the CSF. We observed that an increase in CSF NF‐L levels and reduction in NEUN counting positively correlates with hippocampal atrophy observed via MRI, a result consistent with well‐established observations in AD patients.^30^, ^32^

### 3.5 Microglia are recruited in early areas of tau pathology and correlate with synaptic and neuronal loss

**FIGURE 6:**
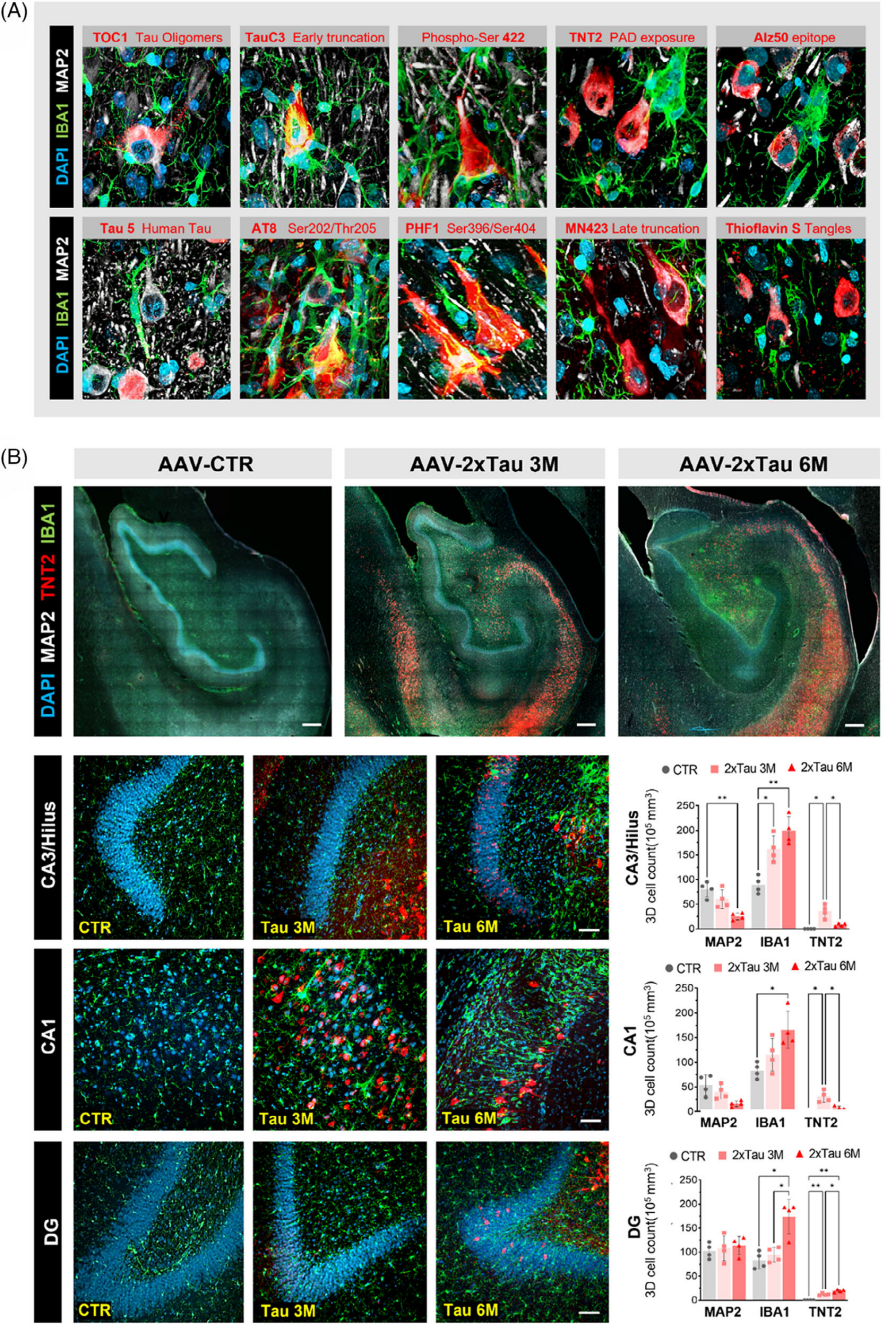
Microglia interacts with early tau pathological markers and drives neuroinflammatory response following 2×Tau delivery. (A) Representative images combining a panel of 10 different markers of tau pathology (red) with microglia marker IBA1 (green), neuronal marker MAP2 (white), and DAPI (blue) across the HF‐ERC region of AAV‐2×Tau injected monkeys at 3 and 6 months. 3D reconstruction analysis indicates microglia is strongly associated with early markers of misfolded tau accumulating in neurons rather than late‐stage markers of the pathology. (B) Fluorescence multilabel microscopy using TNT2 (red), IBA1 (green), and DAPI (blue) was performed and quantified across the CA3/hilus, CA1, and DG regions of all experimental groups. Scale bar: 200 μm and   20 μm. ** *p* < 0.01, ***p < 0.001, ****p < 0.0001, two‐way ANOVA (B), one‐way ANOVA (D), Tukey's post hoc test.

The activation of microglial innate immune response by misfolded and aggregated proteins is believed to contribute to the progression and severity of neurodegenerative processes.^11^, ^34^ In support of that concept, we have previously described an increase in reactive microglia in the HF after 3 months following ERC AAV‐2×Tau delivery.^9^ In this study, we sought to further characterize the interaction between microglia and abnormal tau‐bearing neurons of the ERC and HF across multiple time points after the AAV injection. Toward that end, we combined the panel of 10 different epitopes targeting misfolded tau with IBA1 microglia and MAP2 neuronal markers (Figure 6A; Supplementary Figure 4). Analysis of neurons containing different tau pathology signatures revealed a wide range of microglial responses, ranging from an increased number of ramified microglia surrounding misfolded tau+ cells to the complete enveloping of somas and apical dendrites by IBA1+ cells, as observed for early markers of tau pathology such as TNT2 and Tau C3 (Figure 6A). To further understand how microglia interact with early tau pathology, an IHC combining IBA1 (general microglia marker), MAP2 (neuronal marker), and TNT2, which recognizes a phosphatase‐activating domain (PAD)‐exposed conformational epitope of tau, was performed to unravel how these glial cells interact with early pretangle pathology, sparing late tangles.^26^ As shown in Figure 6B by representative micrographs from all three experimental groups, neuronal reduction is strongly associated with the number and morphology of microglia and the presence of TNT2. In the CA3/hilus region, a sharp decrease in MAP2 was observed when comparing CTR to 6 M 2×Tau animals (68.3% reduction, *p* = 0.0056). In contrast, the IBA1+ population increased approximately 2x fold in both 3 and 6 M groups compared to CTR animals (1.8× increase at 3 M; 2.2 increase at 6 M; *p* = 0.0136, *p* = 0.0027, respectively). The IBA1+ population was also significantly increased across the CA1 and DG regions in 2×Tau 6 M animals compared to the CTR group (97.6% higher in the CA1, *p* = 0.0331; 82.9% higher in the DG, *p* = 0.0185). No TNT2+ cells were detected across all HF fields of CTR animals, and consistent with the hypothesis that aberrant exposure of PAD is an early event in AD progression, significantly higher expression of TNT2 was observed at the 3 M in comparison to 6 M 2×Tau in the CA3 (*p* = 0.0436) and CA1 regions (*p* = 0.0443) (Figure 6B and Supplementary Figures 5 and 6).

To further investigate microglia‐altered profiles, high‐resolution 3D microscopy was performed by combining DAPI, NEUN, and IBA1 with HLA‐DR, one of the genes from the human leukocyte antigen complex known to be upregulated in microglia during neurodegenerative processes.^9^, ^11^, ^35^, ^36^ As shown in the representative images in Supplementary Figure 7A‐B, HLA‐DR colocalizes with IBA1 in several regions across selective parts of the temporal cortex and is exceptionally high in the HF. Little to no expression of HLA‐DR was observed in CTR‐treated animals but was notably higher in layer II of the ERC region of 2×Tau groups, particularly in the 3 M group compared to 6 M animals (Supplementary Figure 7C). Additional analysis of neuron‐microglia interaction after gene delivery was performed by studying 3D microglial morphological alteration and PSD95 puncta density and engulfment by microglia in a protocol extensively published by us and others.^9^, ^11^, ^35^, ^36^ Combining IBA1 with pan‐neurofilament as a neuronal marker and PSD95 as a postsynaptic marker, quantification was performed in the CA3 region across all experimental groups, as shown in Supplementary Figure 7d. Microglial total volume (μm^3^) was significantly higher in the 6 M group compared to CTR (150% higher, *p* = 0.008). Microglia soma shape became amoeboid, and volume (μm^3^) progressively increased between CTR and 3 and 6 M groups (CTR vs. 2×Tau 3 M: *p* = 0.0069; CTR vs. 2×Tau 6 M: *p* < 0.0001; 2×Tau 3 M vs. 2×Tau 6 M: *p* = 0.0047). Analysis of the total PSD95 3D puncta density also showed a similar temporal reduction in synaptic density after AAV‐2×Tau delivery (CTR vs. 2×Tau 3 M: *p* = 0.0087; CTR vs. 2×Tau 6 M: *p* = 0.0002; 2×Tau 3 M vs. 2×Tau 6 M: *p* = 0.0462). Finally, the detection of PSD95 puncta internalized in microglia demonstrated the high microglial engulfment activity following 2×Tau injection in comparison with CTR animals (CTR vs. 2×Tau 3 M: *p* = 0.001; CTR vs. 2×Tau 6 M: *p* < 0.0001).

### 3.6 Neuroinflammation following AAV‐2×Tau delivery is associated with neurodegeneration and local atrophy

**FIGURE 7:**
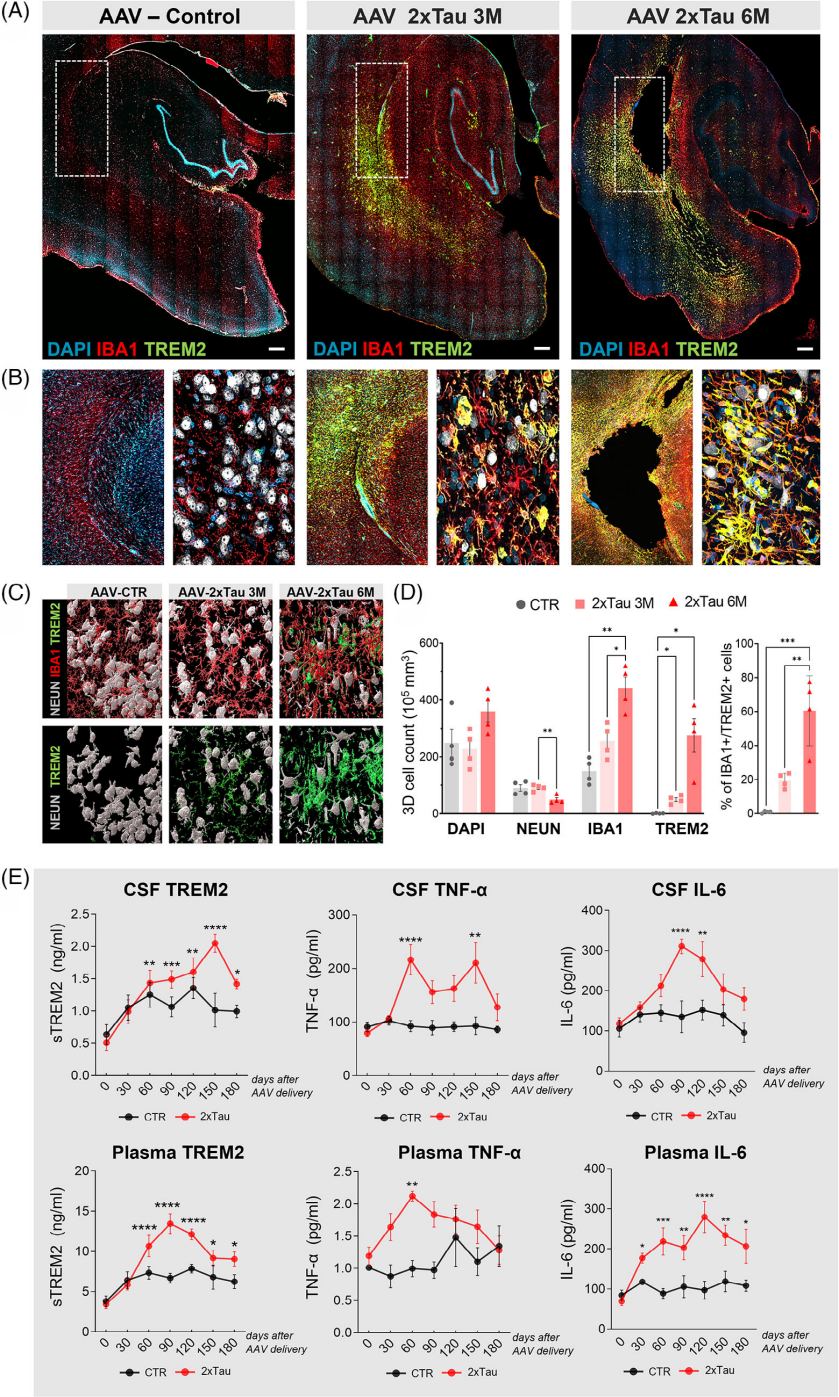
Robust microglial TREM2 expression precedes CA1 hippocampal atrophy. (A) Fluorescence microscopy for the triggering receptor expressed on myeloid cells 2 markers (TREM2, green), the microglial general marker IBA1 (red), and nuclei with DAPI (blue). While TREM2 overexpression precedes tissue neurodegeneration, there is a clear pattern of spatial colocalization between high TREM2 expression and histological damage. (B) Higher magnification of the boxed area in (A) including NEUN (white). (C,D) Representative images of the selected hippocampal areas analyzed and quantification of the 3D cellular density for each cellular marker investigated. A significant reduction in the NEUN+ population occurs concomitantly with a robust increase in total IBA1+ population recruitment. (E) The increase in TREM2 expression by microglia in the brain is associated with an increase in soluble TREM2 levels in the CSF and plasma. Additional analysis of the proinflammatory cytokines TNF‐alpha and IL‐6 was performed in the CSF and plasma across all experimental groups. Scale bar: 500 μm *p < 0.05 **p < 0.01, ***p < 0.001, ****p < 0.0001, two‐way ANOVA, Tukey's post hoc test.

During neurodegenerative processes, ramified microglia progressively shift from a homeostatic profile to a disease‐associated state (DAM),^37^ in which several genes, including TREM2, are upregulated.^38^ To gain insight into the role of microglia in the tau‐driven pathology observed in treated monkeys, we combined a TREM2 marker with an IBA1 microglial general marker and DAPI for total cell counting. Representative 3D reconstructions of the HF and ERC region across the different animal groups exemplify the highly selective TREM2 expression increase observed adjacent to the CA1 hippocampal area (Figure 7A). Higher magnification imaging of the HF (Figure 7B) and 3D segmentation and quantification confirmed our qualitative observations (Figure 7C,D; Supplementary Figure 8). On average, the total DAPI counts in the selected region showed a trend toward an increase of 44% in 6 M animals in comparison with the CTR, despite not reaching significance (*p* = 0.0824). In contrast, NEUN density was reduced by half at 6 M compared to the CTR group (CTR vs. 2×Tau 6 M, *p* = 0.0038), showing an active neurodegenerative process that is accompanied by a progressive increase in local microglia recruitment. Furthermore, there is an increase in the total number of TREM2+ cells that happens at 3 months after 2×Tau injection (CTR vs. 2×Tau 3 M, *p* = 0.0131) which is substantially increased at 6 months (CTR vs. 2×Tau 6 M, *p* = 0.0374). This increase in TREM2 immunosignal results in a considerably higher proportion of IBA1+ cells colocalizing with TREM2 at 6 M compared to 3 M (% of IBA+/Trem2+ cells: 19% at 3 M, 60% at 6 M; Figure 7C,D). Critically, no relevant TREM2 expression was detected in the animals injected with the AAV‐CTR construct, suggesting that a selective local CA1 TREM2‐driven inflammatory response results from misfolded tau propagated from the injected ERC to the HF region (Figure 7D).

We also investigated whether brain inflammation could be observed in the CSF and plasma of AAV‐infected monkeys. We detected a progressive increase in soluble TREM2 levels following AAV‐2×Tau delivery in both CSF and plasma, but not in the AAV‐CTR injected animals, reaching statistical significance 90 days after 2×Tau injection (*p* = 0.0014, Figure 7E). Additionally, soluble levels of proinflammatory cytokines TNF‐α and IL‐6 were also increased following the weeks after AAV‐2×Tau delivery. The soluble levels of TNF‐α presented a dual transient increase in the CSF, with the highest increase in 2×Tau animals 60 days after injection (*p* = 0.0041). Plasma levels of TNF‐α were also increased 60 days following injection of 2×Tau animals (*p* = 0.005). IL‐6, another cytokine highly associated with neuroinflammatory processes occurring in AD,^39^ reached the highest levels in the CSF 90 days after the 2×Tau delivery (*p* = 0.0005), while levels remained elevated in the plasma throughout the 180‐day period (Figure 7E).

### 3.7 Microglia and astrocytes coordinate glial response to tau pathology, targeting different stages of tangle formation

**FIGURE 8:**
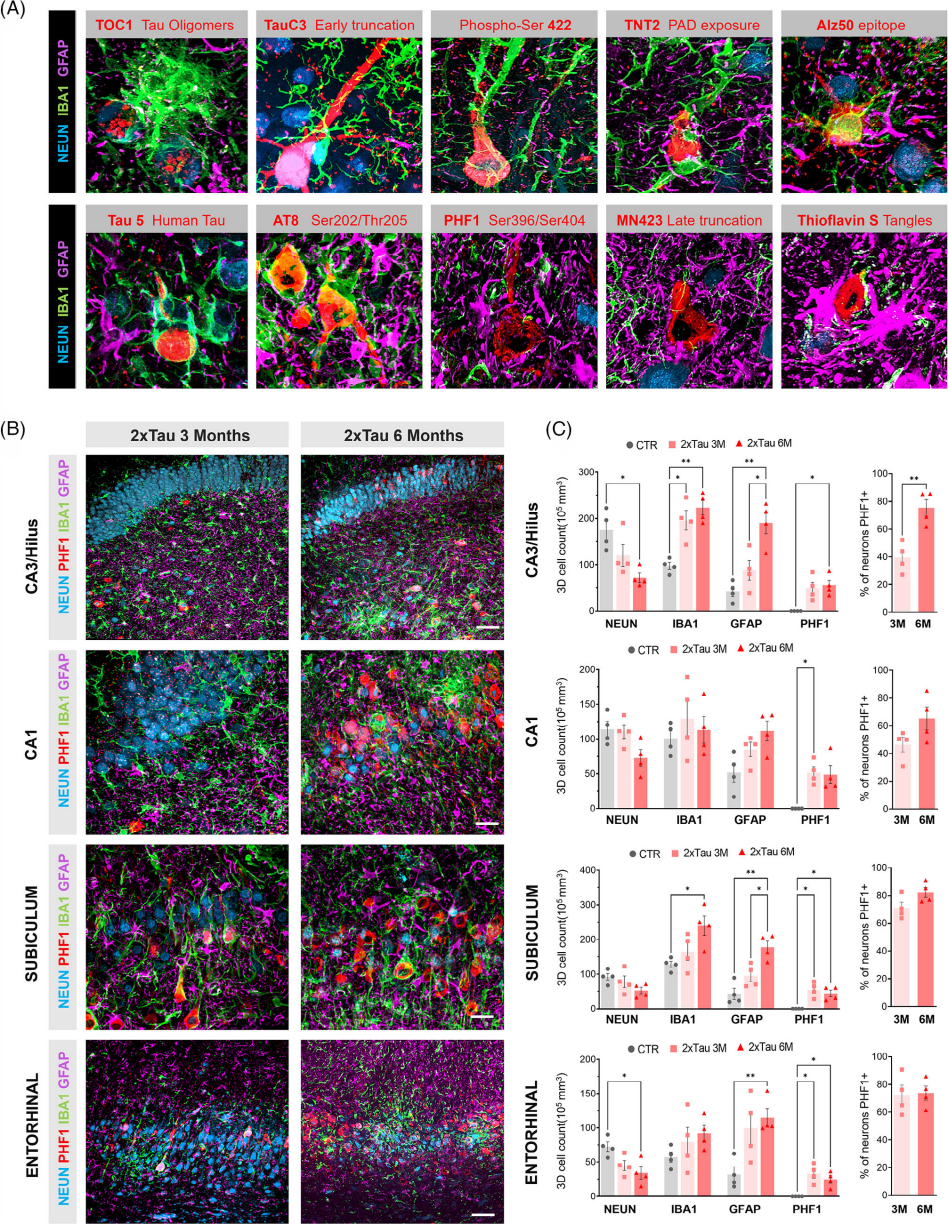
Tau pathology progression is associated with microglia and astrocyte‐driven complementary neuroinflammatory responses. (A) Fluorescent photomicrographs illustrating the relationship between glial cells and abnormal tau‐containing cells in the ERC‐HF complex of 3 and 6 M AAV‐2×Tau animals. General markers for neurons (NEUN, blue), microglia (IBA1, green), and astrocytes (GFAP, purple) were combined with the 10 different tau pathology markers previously investigated in this study (red). (A) While microglial interactions predominate for neurons containing early epitopes of tau pathology, astrocytes become the driving force when paired‐helical structures have formed, and neurons progress into the middle‐to‐late stages of the pathology. (B) To further investigate glial cell interaction with tau late‐stage pathology, quadruple staining was performed using PHF1 antibody (Ser396/Ser404) combined with NEUN, IBA1, and GFAP across the ERC‐HF region. (C) Quantification of the total 3D cell density of each cell type across hippocampal regions. The total percentage of neurons expressing PHF1 was calculated based on the total NEUN+ population in the CA3/Hilus, CA1, SUB, and left ERC (C) and reflects a shift in the tangle formation, microglia and astrocyte recruitment across the three and six time‐points period. Scale bar: 50 μm, *p < 0.05 **p < 0.01, two‐way ANOVA, Tukey's post hoc test.

Astrocytes are glial cells also involved in misfolded tau spreading in AD and other tauopathies.^40^ Notably, higher expression of GFAP is known to be a marker of neuroinflammatory response driven by astrocytes in the AD brain.^41^ We sought to investigate how microglia and astrocytes potentially interact with misfolded tau in the HF‐ERC of 2×Tau animals. Therefore, we combined general markers for neurons (NEUN), microglia (IBA1), and astrocytes (GFAP) with the panel of 10 different markers of tau pathology used in this study previously. As shown in Figure 8A and Supplementary Figure 9, in the early stages of misfolded tau formation, microglia are the primary component driving the neuroinflammatory response, directly interacting with neurons expressing tau oligomers (TOC1), early tau truncation (TauC3), TNT2, and early phosphorylation (Alz50). As the neurodegenerative process progresses, astrocytes appear to take a significant role in coordinating the neuroinflammatory response and are found chiefly directly interacting with neurons expressing later fibrillary tangle markers such as PHF1 (late phosphorylation), MN423 (late truncation), and ThioS (tangles) (Figure 8A). In several instances, astrocytes were also found interacting with ameboid microglia‐degraded neuron complexes, suggesting a debris‐clearing role for astrocytes at advanced stages (Figure 8A,B; Supplementary Figure 10).

To further investigate potential glial‐neuron interactions in the presence of misfolded pathological tau, we performed a quadruple staining combining the late‐stage epitope PHF1 with NEUN, IBA1, and GFAP. We quantified the 3D cell density of each cell type across the HF‐ERC regions (Figure 8B,C; Supplementary Figures 11 and 12). We found a progressive temporal decrease in the number of neurons across all areas analyzed, correlating with an increase in the microglial and astrocytic populations. The CA3/hilus and ERC were the regions with the most pronounced reductions in the neuronal population, with 59% and 53% reduction observed, respectively, in comparison with AAV‐CTR animals (*p* = 0.0207, CA3; *p* = 0.0433, ERC). In the CA1 and SUB, the neuronal density was reduced by 36% and 42%, respectively, in the 6 M group compared with CTR. Notably, in the ERC, PHF1 expression is already high at the 3 M time‐point (72%) and remains high at the 6 M time‐point (73%), contributing to the robust neuronal loss observed in this region. Similarly, SUB also presents a high degree of colocalization between NEUN and PHF1, reaching 71% at 3 M and increasing to 82% at the 6 M group. CA3 and CA1 regions presented a slower but consistent progression in PHF1 expression, increasing from 40% and 47% at 3 M to 75% and 65%, respectively, in the 6 M group. The robust neuronal population reduction was accompanied by a significant increase in microglia and astrocyte local recruitment (Figure 8C). In CA3, the microglia population doubled at 3 M and remained high at 6 M in comparison with CTR (*p* = 0.0278, 3 M; *p* = 0.0030, 6 M). An increase in microglial local recruitment was also observed in the SUB 6 months after AAV delivery (*p* = 0.0495). In addition to microglia, a pronounced local recruitment of astrocytes was observed in all the regions analyzed, notably in direct interaction with PHF1+ neurons. CA3 presented the highest increase in the GFAP+ population, particularly later in the pathology, reaching a 2× fold increase at 3 M and 4.8× fold at 6 M compared with CTR (*p* = 0.0089, 6 M). Interestingly, we also detected a difference in astrocytic population in the CA3 region between the two‐time points after the 2×Tau injection analyzed in CA3 (2×Tau 3 M vs. 2×Tau 6 M, *p* = 0.0416). A similar profile was also observed in the SUB region (CTR vs. 6 M, *p* = 0.0034; 2×Tau 3 M vs. 2×Tau 6 M, *p* = 0.0332). Finally, a difference in the total number of astrocytes was also observed later in the disease in the ERC region (CTR vs. 2×Tau 6 M, *p* = 0.0073) and the CA1 (2× fold increase) despite the latter not reaching statistical significance (Figure 8C).

## 4 DISCUSSION

AD is increasingly becoming one of the most burdensome diseases of the century, affecting over 43 million people globally and expected to impact over 150 million individuals worldwide by 2050.^42^ These numbers highlight both the urgent need for disease‐modifying therapies and the slow progress of amyloid‐beta‐targeting strategies, igniting interest in tau‐oriented approaches in recent years.^43^ This interest is fueled by *post mortem* and in vivo studies, including fluid biomarkers and PET imaging, which consistently show a relationship between tau pathology and cognitive impairment in the AD clinical spectrum.^44^, ^45^, ^46^ Mounting evidence suggests that tau pathology propagates between interconnected brain areas via conformational changes in a prion‐like manner, leading to impaired neuronal function, synaptic dysfunction, and neuronal loss that better correlate with the cognitive deficits observed in AD typical presentations.^9^, ^10^, ^47^, ^48^, ^49^, ^50^, ^51^, ^52^ The strict neuroanatomical progression of tau pathology, however, makes it particularly challenging to study in experimental models that do not reproduce the connectivity of the human brain, making the development of NHP models an important step in the field.^6^, ^53^

Toward that goal, we sought to develop a rhesus‐based tauopathy model that reproduces the AD‐associated propagation of pathological tau through the ERC connectome. Murine studies have shown that neocortical intracerebroventricular delivery of an AAV containing the P301L/S320F double‐mutant leads to an accelerated tau pathology phenotype characterized by abundant tau phosphorylation, tangle formation and memory impairment,^9^ prompting us to pursue a similar approach. However, to better leverage the monkey neuroanatomical complexity and better mimic the progression of tau pathology, we opted to perform injections of the viral vector into the ERC and to use adult animals, as opposed to neonates. As expected, this approach was successful in recapitulating multiple aspects of AD, including (1) induction of misfolded tau, both locally (ERC) and throughout interconnected regions; (2) coaptation of endogenous tau, leading to templating of macaque tau into pathological forms; (3) induction of neuroinflammation; and (4) alterations in AD core biomarkers in CSF and blood.^9^ With a single time‐point, however, several questions were outstanding regarding the temporal progression of the pathology in this model and its ability to reflect that progression through different biomarker modalities.

The present study addresses these questions and significantly expands the model's scope by including a 6‐month time‐point in addition to a new 3‐month cohort and by extending the biochemical characterization with several tau epitope antibodies. This further characterization of the model has revealed that neuronal pathology continues to be exacerbated aggressively from 3 to 6 months, as evident by a dramatic increase in tau epitopes associated with more advanced tau pathology, an increase in end‐stage tangles, and neuronal death at 6 months that was not apparent at 3 months. Furthermore, we observed an expansion of the spatial distribution of tau epitopes throughout the ipsilateral temporal lobe and the contralateral hippocampus between 3 and 6 months, suggesting there is an active but delayed induction mechanism through intra‐ and interhemispheric connections. This further strengthens the idea that the spatiotemporal pathology progression in the model is driven primarily by the transport of pathological seeds through areas connected to the ERC and hippocampus, as hypothesized for AD.^45^

Critically, we also saw an increase in the templating of endogenous tau by pathological tau at 6 months, indicating that the spread of tau pathology observed between 3 and 6 months is mediated by the continued and exacerbated interaction between endogenous rhesus monkey tau and the initial pathological tau transcribed and translated from the human mutated tau. This rapid progression of tau‐based pathology occurred without any manipulation of Aβ oligomers (AβOs), whereas a recent paper demonstrated that administration of AβOs exacerbated more moderate tau‐based pathology induced by administration of tau‐seeds from human AD brains.^54^ Importantly, the time frame and nature of the tau manipulation were very different across these two studies, with the greater propagation over a shorter timeframe making our model more amenable for therapeutic testing. The potential for AβOs to exacerbate tau‐based pathology in diverse NHP models of AD, however, remains an important issue for further investigation.

The more comprehensive approach taken in the present work has also revealed novel insights into tau‐associated neuroinflammation. We observed a fundamental difference in the nature of neuron‐glia interactions between the two time‐points of this study. At 3 months, we observed extensive interactions between pathologic tau and microglia, as neurons containing the earliest reflections of tau biochemical alterations were most strongly associated with microglia displaying both morphological (amoeboid morphology) and chemical (TREM2) signs of activation. However, as late‐stage tau pathology epitopes become more prominent at 6 months, the inflammatory response shifts to one mediated primarily by astrocytes, which envelop both neurons and dysmorphic microglia. Taken together, these observations suggest that, independent of amyloid pathology, pathologic tau induces a two‐phase neuroimmune response: the first phase is characterized by cytoskeletal disruption concomitant with high microglial activity, and the second phase is marked by frank neuronal loss coupled with high levels of astrogliosis. These results are relevant to therapeutic interventions aimed at neuroinflammation, as the modulation of microglial activity is more likely to impact the course of neurodegeneration, while astrocytes appear to become involved after the degenerative cascade is at an advanced stage.

The new 6‐month group has also allowed for a more comprehensive analysis of in vivo biomarkers that successfully track pathological progression in live animals. Central tau pathology was fully reflected in CSF and plasma biomarkers for tau, neurodegeneration, and neuroinflammation, all remaining elevated for the entire duration of the experiment. Furthermore, in addition to fluid biomarkers, both MRI and PET imaging showed hippocampal atrophy and detectable tau signal, respectively, which correlated with changes in clinically translatable imaging and fluid biomarkers, suggesting that this model may be amenable to detecting effects of treatment on these markers in relatively small studies. The rates of change of hippocampal tau PET SUVr (increases in the range ∼0.1‐0.5 over 6 months) and hippocampal GM loss (decreases in the range ∼5‐20% in 6 months) are both substantially greater than those observed in sporadic human AD. Tau PET in the hippocampus per se is challenging to measure with some PET tracers due to off‐target binding in the choroid plexus but, as a point of comparison, longitudinal change in tau SUVr in an ROI predominantly containing the fusiform gyrus as measured using the tracer [^18^F]flortaucipir has been reported as up to 0.3 SUVr units/y (mean SUVr 0.074 units/y) in an amyloid positive, cognitively impaired population.^55^ Similarly, the mean decrease in hippocampal volume in mild AD populations is typically in the range of 3% to 5% annually.^56^, ^57^, ^58^ These suggest that the progression of pathology in the 2×Tau NHP model is more aggressive and rapid than sporadic AD in humans, increasing the statistical power to detect a slowing of these progressive imaging biomarkers in a manageable time window and sample size.

In combination, both fluid and imaging biomarker alterations were strongly supported by our histological observations, closely correlating with the biochemical, spatial, and temporal progression of tau pathology. While the rate of change of fluid biomarkers was highest in the first 90 days, often stabilizing or decreasing after this point, in vivo, imaging was more closely associated with the severe hippocampal pathology and neuroinflammation observed at the 6‐month time‐point. These observations suggest that fluid biomarkers are more sensitive metrics for the early events in the tau pathological cascade when tau‐targeting therapies may be most efficient at preventing disease progression. On the other hand, in vivo imaging may be more effective as a proxy of disease severity, when neuronal death and late‐stage tangle formation have already occurred. Furthermore, in vivo imaging in our model did not accurately reflect the incipient pathology observed in the temporal neocortex, suggesting that seeding and pathology dispersion likely precede a positive PET signal in these areas.

Despite the significant results obtained with this model, the experimental approach chosen has several caveats that merit discussion. First, we opted to only employ female animals in this study. This choice was made to focus precisely on the most vulnerable part of society since most Americans living with AD are women,^59^ and there is a large body of literature on the relationship between the female hormonal milieu, synapse loss, and aging.^60^, ^61^, ^62^ Second, while this model replicates the neuroanatomical progression of tau pathology seen in AD, this process is occurring in the absence of amyloid pathology. Future studies combining the present approach with either naturally occurring or induced amyloid pathology will be important to shed light on the relationship between tau and amyloid cascades in AD. Third, the double mutations used in this study are associated with frontotemporal dementia and Pick's disease, rather than AD. This is a necessary compromise, as there are no MAPT mutations strictly associated with AD, and murine studies have suggested that nonmutated tau overexpression leads to slow‐progressing, limited pathology.^10^ Finally, while this model will be highly useful to explore tau‐based mechanisms of neurodegeneration, it is important to underscore that the use of mutated tau, the lack of amyloid involvement, and the age of our animals limit the direct use of this model to explore sporadic, age‐associated neurodegenerative changes.

In summary, and as shown in Supplementary Figure 13, following the AAV‐2×Tau injection, our multifaceted approach has shown that there is a long window, likely to extend at least from 2 to 6 months, when pathology is rapidly progressing on several measurable levels. Through the in vivo and microscopic dissection of the hypothesized temporal sequence presented here, even a subtle arrest of this process through therapeutic intervention should be readily observable and quantified, providing a powerful opportunity to test therapeutics that address different stages of the pathologic process, including the very early events that precede the irreversible behavioral decline. Furthermore, the experimental approach delineated here can be used to quantify the progression of intraneuronal degenerative mechanisms and the associated neuroinflammatory processes with great precision, which we are confident can be further explored to expand our understanding of neurodegenerative processes.

## AUTHOR CONTRIBUTIONS

D.B. contributed to the experimental design, execution of histological and biofluid analyses, overall data analysis and interpretation, and manuscript preparation. G.B.D. contributed to the data analysis and interpretation, as well as manuscript preparation. S.O. contributed to the tissue collection and the execution of experiments. B.H. and A.J.C. contributed to the in vivo imaging acquisition, including PET and MRI experiments, data interpretation, and manuscript preparation. Y.C. and S.M. contributed to the neurosurgical procedures and stereological analyses. P.C. made the viral vector employed in this work available and participated in the data interpretation and manuscript preparation. N.M.K. contributed multiple primary antibodies employed in this study and participated in the data interpretation and manuscript preparation. J.K. contributed to the experimental design, performed the neurosurgeries, oversaw stereological experiments, and participated in data interpretation and manuscript preparation. J.H.M. and A.S. contributed to the overall study planning and experimental design, data analysis and interpretation, and manuscript preparation.

## CONFLICT OF INTEREST STATEMENT

The authors from UC Davis, Arizona State University, University of Florida, Michigan State University, and the Rush University Medical Center have no competing interests to report. The current and former Takeda authors, although receiving salary and stock options from Takeda, have no competing interests to report. Author disclosures are available in the supporting information.

## CONSENT STATEMENT

Human consent does not apply to this study.

## Supporting information

Supporting information

Supporting information
