# Supplementary material for: Temporal progression of tau pathology and neuroinflammation in a rhesus monkey model of Alzheimer's disease
Source: Alzheimers Dement. 2024 Jun 21;20(8):5198–219. doi: 10.1002/alz.13868 (PMC11350056; doi:10.1002/alz.13868)
Supplement: Supplementary file 1 — Supporting information [file ALZ-20-5198-s001.docx]

**Supplementary Information for**

**Temporal Progression of Tau Pathology and Neuroinflammation in a Rhesus Monkey Model of Alzheimer's Disease**

Authors: Danielle Beckman^1^, Giovanne B Diniz^1^, Sean Ott^1^, Brad Hobson^1,2^, Abhijit J. Chaudhari^1,2^, Scott Muller^3^, Yaping Chu^3^, Akihiro Takano^4^, Adam J. Schwarz^4^, Chien-Lin Yeh^4^, Paul McQuade^4^, Paramita Chakrabarty^5^, Nicholas M. Kanaan^6^, Maria S. Quinton^4^, Arthur A. Simen^4^, Jeffrey H. Kordower^3,7^, John H. Morrison^1,8^

Affiliations:

**1.** California National Primate Research Center, University of California Davis; Davis, California, USA

**2.** Department of Radiology, School of Medicine, University of California, CA, Sacramento, USA

**3.** ASU‐Banner Neurodegenerative Disease Research Center, Arizona State University, Tempe, Arizona

**4.** Takeda Pharmaceutical Company Ltd, Cambridge, MA, USA

**5**. Department of Neuroscience, Center for Translational Research in Neurodegenerative Disease, University of Florida, Gainesville, Florida, USA

**6.** Department of Translational Neuroscience, College of Human Medicine, Michigan State University, Grand Rapids, Michigan, USA.

**7.** Department of Neurological Sciences, Rush University Medical Center, Chicago, Illinois, USA

**8.** Department of Neurology, School of Medicine, University of California Davis; Davis, California, USA.

^*^**Correspondence**
John H. Morrison, University of California Davis, California National Primate Research Center, UC Davis, One Shields Avenue. Davis, CA 95616, USA.
Email: [jhmorrison@ucdavis.edu](mailto:jhmorrison@ucdavis.edu)

**
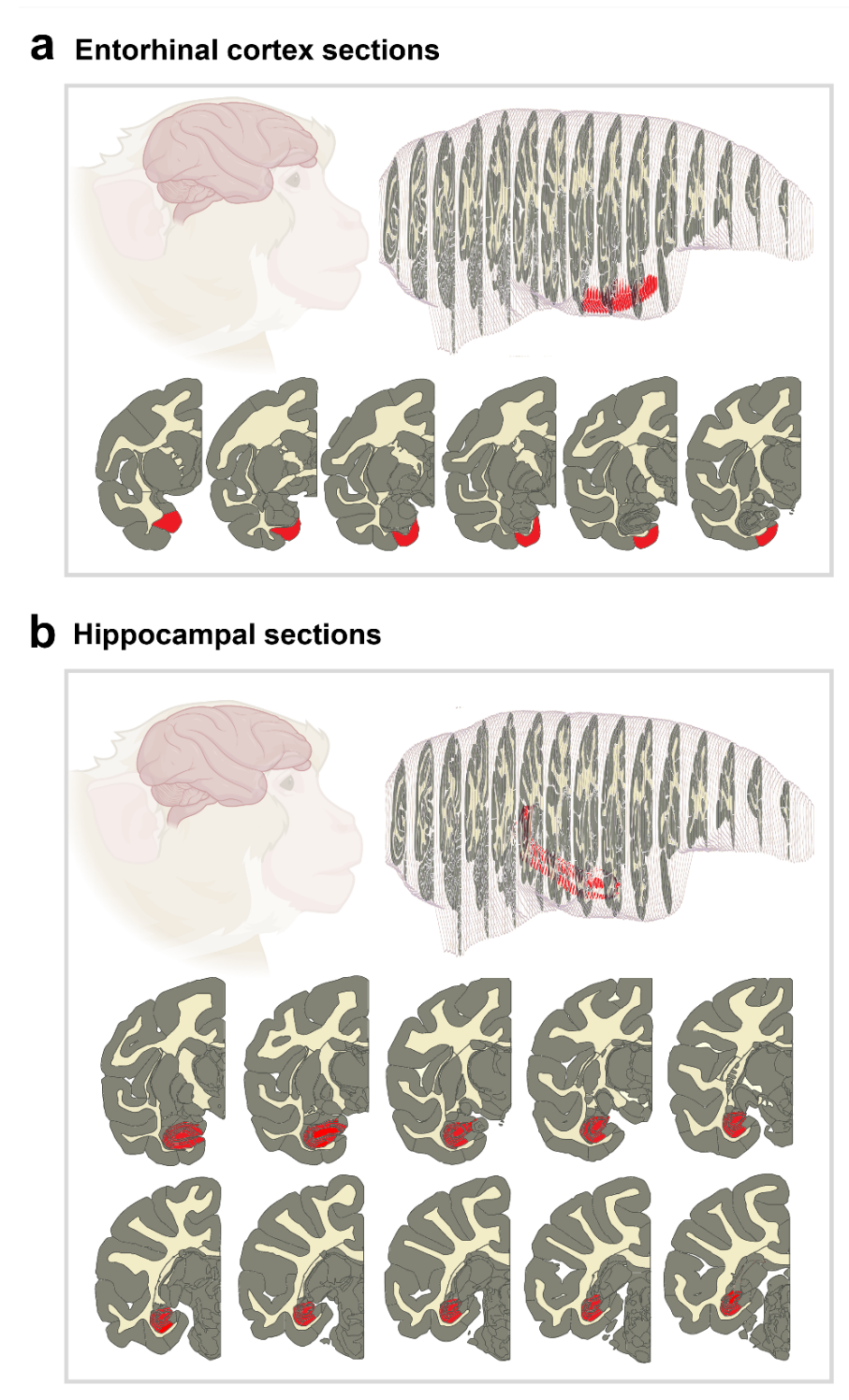
Supplementary Figure 1 – Diagrammatic representation of the anteroposterior extension of brain areas analyzed in this study.** Quantitative histological analyses presented in this study were performed on coronal sections that included the **(a)** entorhinal cortex (ERC) and **(b)** hippocampus (Hip), as highlighted in red in the figure. Adapted from Dubach and Bowden (2009) and Rohlfing et al. (2012).


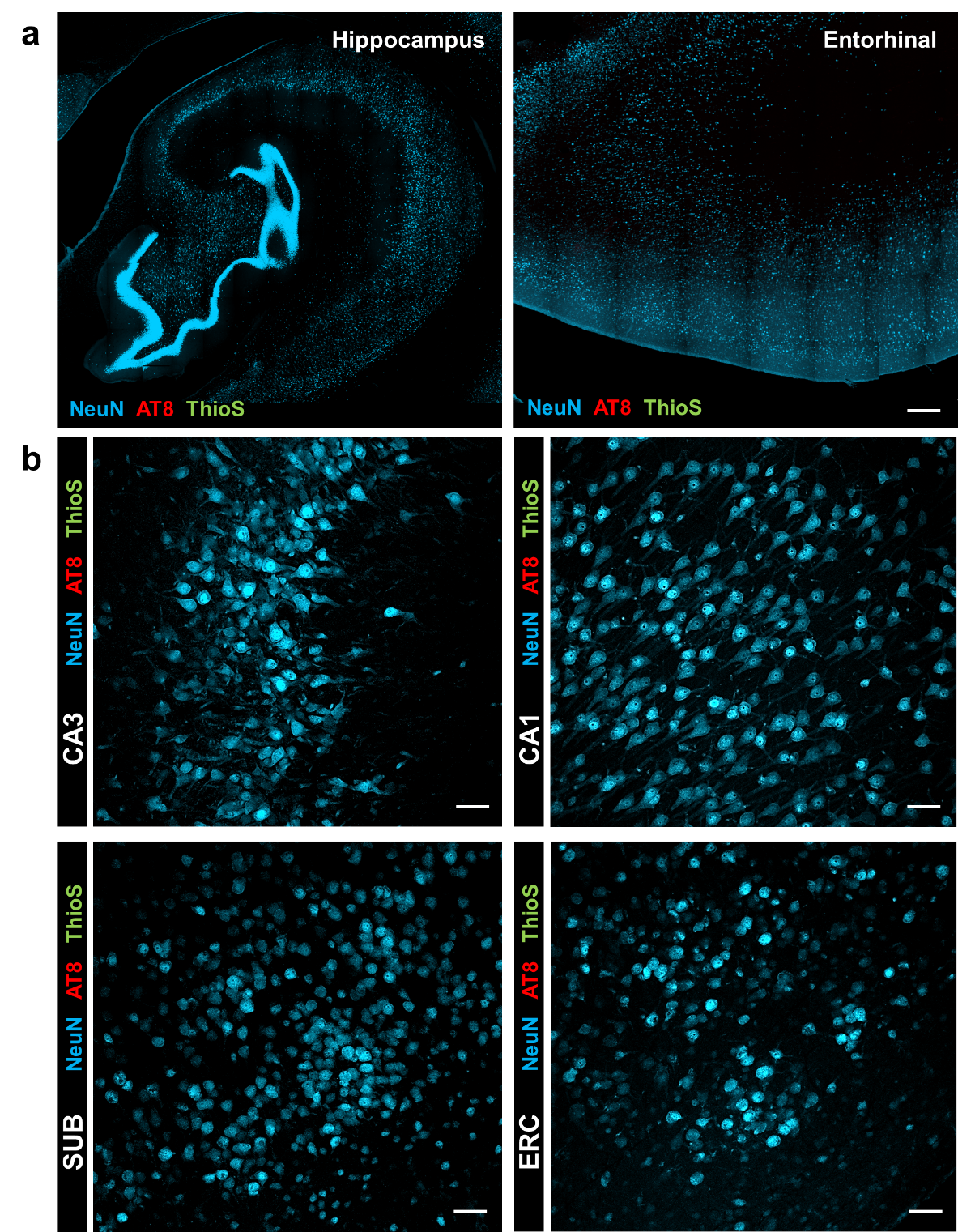


**Supplementary Figure 2 – No hyperphosphorylated tau or neurofibrillary tangles detected in control (empty vector) injected animals.** Similar to AAV-2xTau animals, the hippocampus and entorhinal region of AAV-CTR animals were also analyzed. **(a)** No AT8 or ThioS neurons were observed in none of the regions across both experimental control groups, 3- and 6-months groups. **(b)** Higher resolution images of the CA3, CA1, Subiculum (SUB) and injected entorhinal (ERC) illustrate that neuronal expression is preserved in these regions following 3 or 6 months after the empty vector delivery.

**
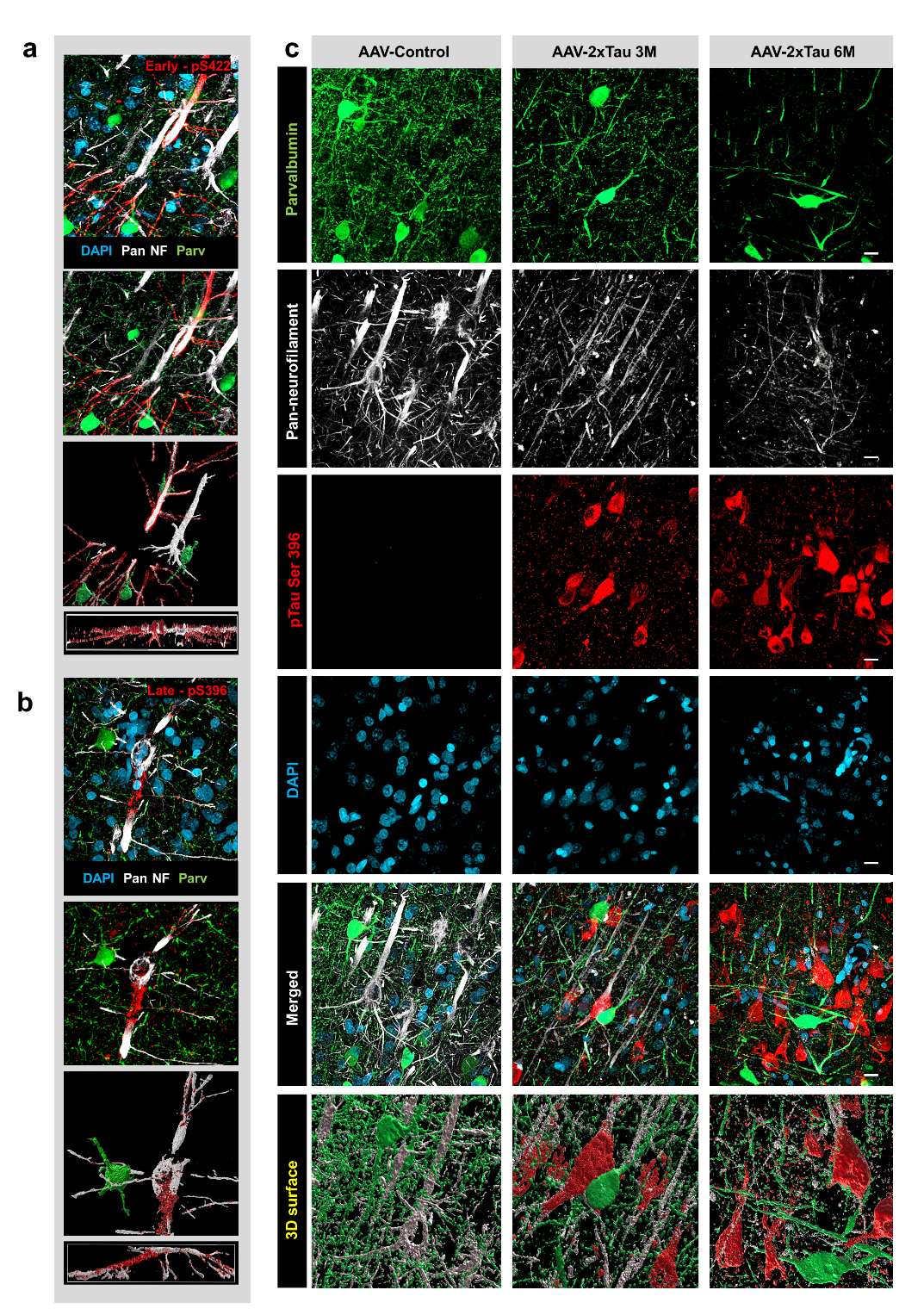
**

**Supplementary Figure 3 – Virally-induced Tau overexpression correlates with cytoskeletal breakdown in large pyramidal neurons.** Photomicrographs and 3D reconstructions from frontal sections of AAV-2xTau rhesus macaques subject to immunohistochemistry for the neuronal cytoskeletal protein neurofilament light (Pan NF – white), parvalbumin (Parv – green), and the pathology-associated Tau epitopes pS422 and pS396 (red), and counterstained for nuclei visualization (DAPI – blue). Phospho-serine 422, an early marker of Tau pathology, can be observed in the soma but also in the dendritic processes of neurons, where it colocalizes with Pan NF, revealing a similar intracellular misplacement of Tau as observed in AD pathology **(a)**. Phospho-serine 396, a later marker of Tau pathology, poorly colocalizes with Pan NF, appearing in areas of disrupted morphology where Pan NF is absent. This suggests that when this posttranslational modification is present, there is a breakdown of the underlying cytoskeleton, and this effect is more pronounced at 6 months than at 3 months **(b,c)**. Within areas of phosphorylated Tau expression, there is minimal colocalization between pTau and parvalbumin, suggesting the model reproduces the neuronal vulnerability of large pyramidal neurons to Tau pathology. At the same time, GABAergic interneurons are less likely to display pathological Tau **(c)**. These results strongly suggest that the AAV-Tau model extensively reproduces essential properties of Tau pathology observed in AD patients and other animal models. Scale bar: 20 µm.

**
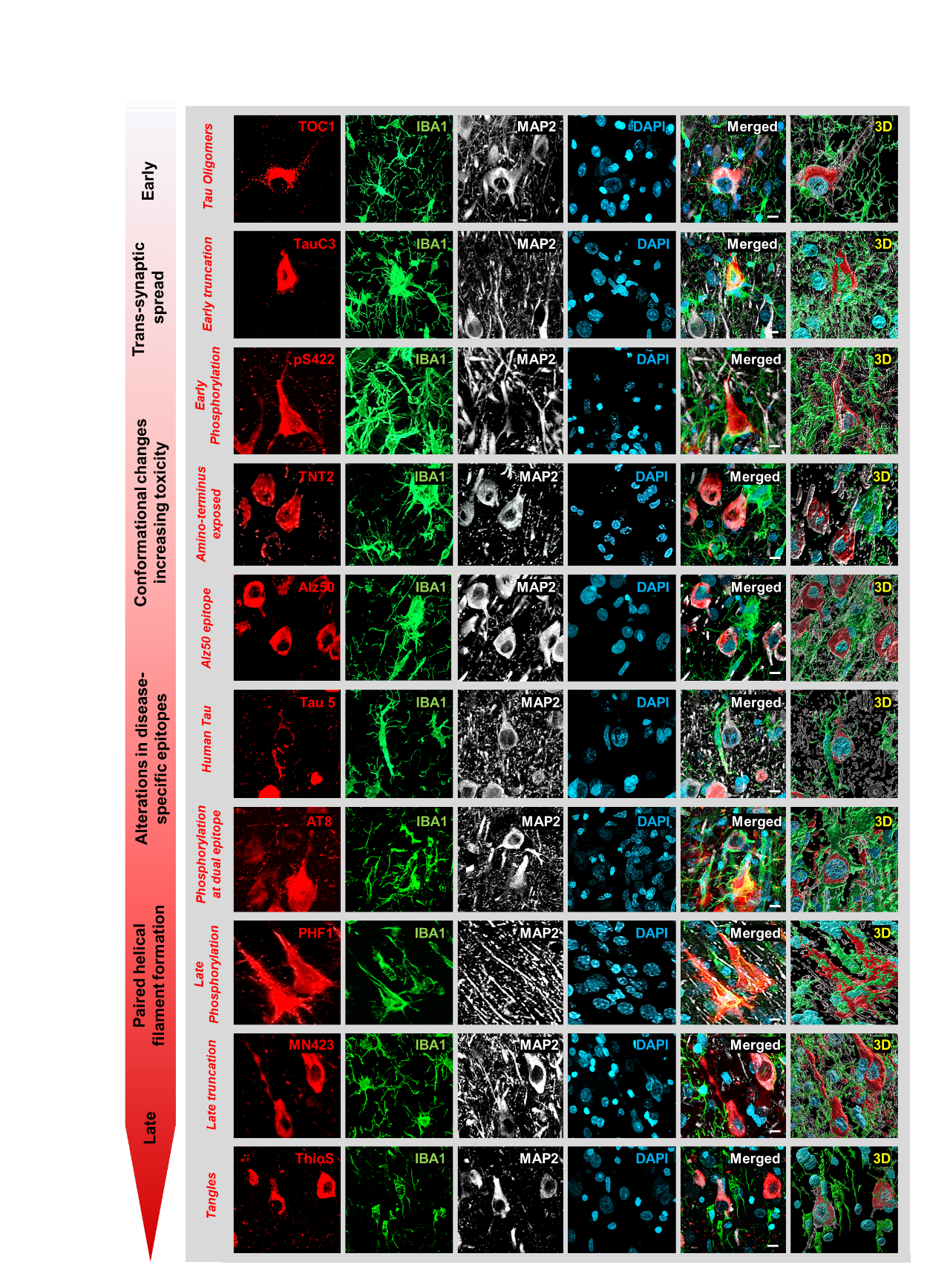
**

**Supplementary Figure 4 – Microglia adopt amoeboid morphology near Tau-containing neurons, often adopting the shape of the somas they contact.** Separate channel visualization and 3D reconstructions of the photomicrographs in **Figure 6a** illustrate the engulfment of pathological Tau-containing neurons by neighboring microglia in 3M and 6M AAV-2xTau animals. Separate channels correspond to the neuronal cytoskeletal protein MAP2 (white), microglia (IBA1 - green), neurons (DAPI – blue), and multiple Tau epitopes (red). Tau epitopes are organized from top to bottom based on their relative order of appearance and role played at distinct stages of Tau pathology, from earlier markers on the top to late-stage markers on the bottom, as indicated by the graphical elements on the left. Specific Tau epitopes strongly correlate with an enveloping microglia morphology, such as TauC3, pS422, AT8, and PHF-1, suggesting a potential epitope-dependent microglial response. Microglial enveloping was also associated with a decrease in MAP2 expression. Scale bar: 5 µm.

**
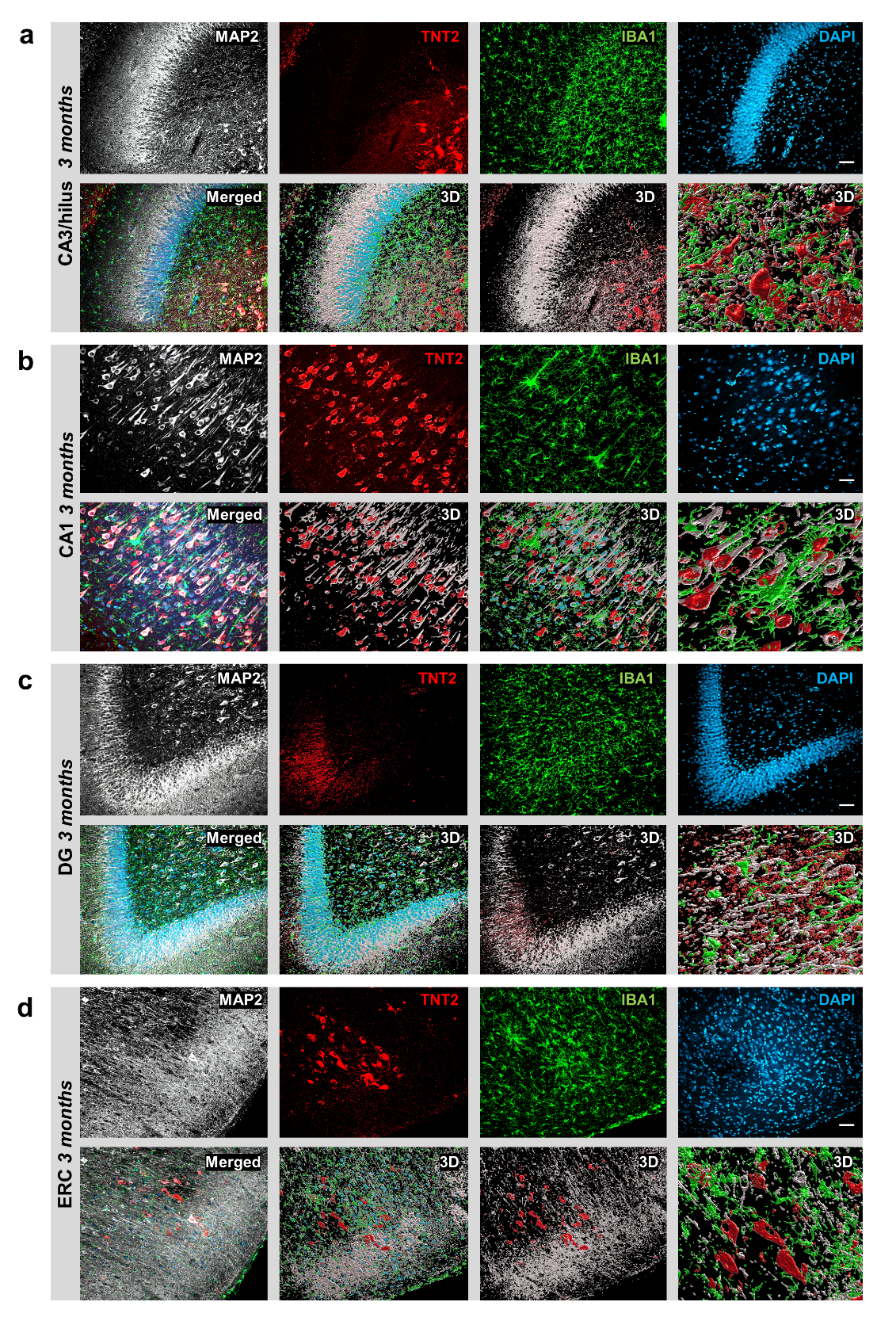
**

**Supplementary Figure 5 – Association between microglia and TNT2 in the hippocampus and entorhinal cortex of AAV-2xTau animals at 3 months after injection.** Separate channel visualization and 3D reconstructions of photomicrographs present in **Figure 6c** highlight the expression of the neuronal cytoskeletal protein MAP2 (white), the Tau epitope TNT2 (red), microglia (IBA1 – green), and nuclei (DAPI – blue) in the CA3/hilus **(a)**, CA1 **(b)**, dentate gyrus **(c)**, and entorhinal cortex **(d)**. Three-dimensional reconstructions, such as the ones displayed here, were used to perform morphometric quantifications presented in the study. Notice the close association between IBA1+ cells and MAP2/TNT2+ neurons in CA1. Scale bar: 25 µm.


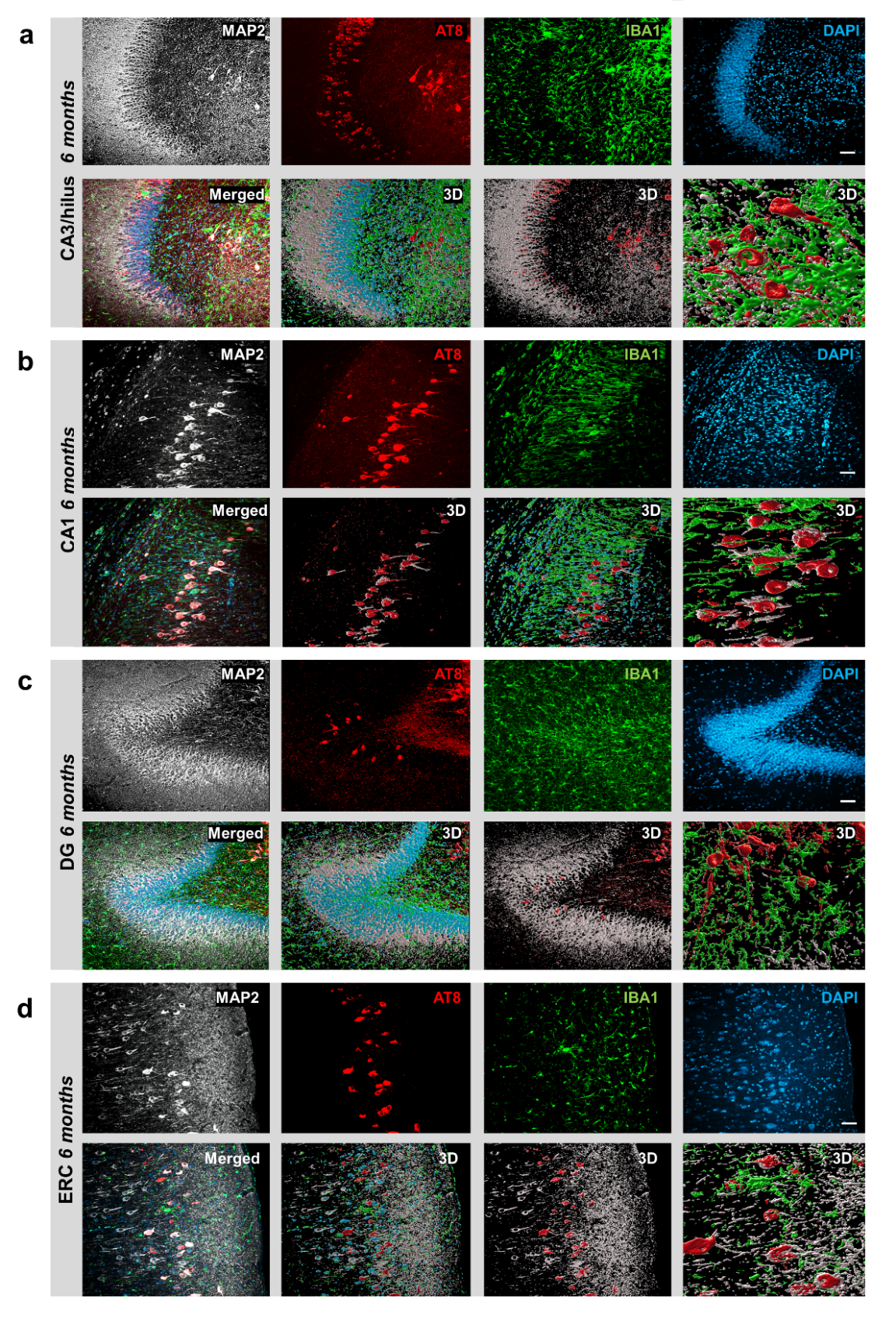


**Supplementary Figure 6 – Association between microglia and TNT2 in the hippocampus and entorhinal cortex of AAV-2xTau animals 6 months after injection.** Separate channel visualization and 3D reconstructions of photomicrographs present in **Figure 6c** highlight the expression of the neuronal cytoskeletal protein MAP2 (white), the Tau epitope TNT2 (red), microglia (IBA1 – green), and nuclei (DAPI – blue) in the CA3/hilus **(a)**, CA1 **(b)**, dentate gyrus **(c)**, and entorhinal cortex **(d)**. Three-dimensional reconstructions, such as the ones displayed here, were used to perform morphometric quantifications presented in the study. Notice the close association between IBA1+ cells and MAP2/TNT2+ throughout the hippocampal formation. Scale bar: 25 µm.


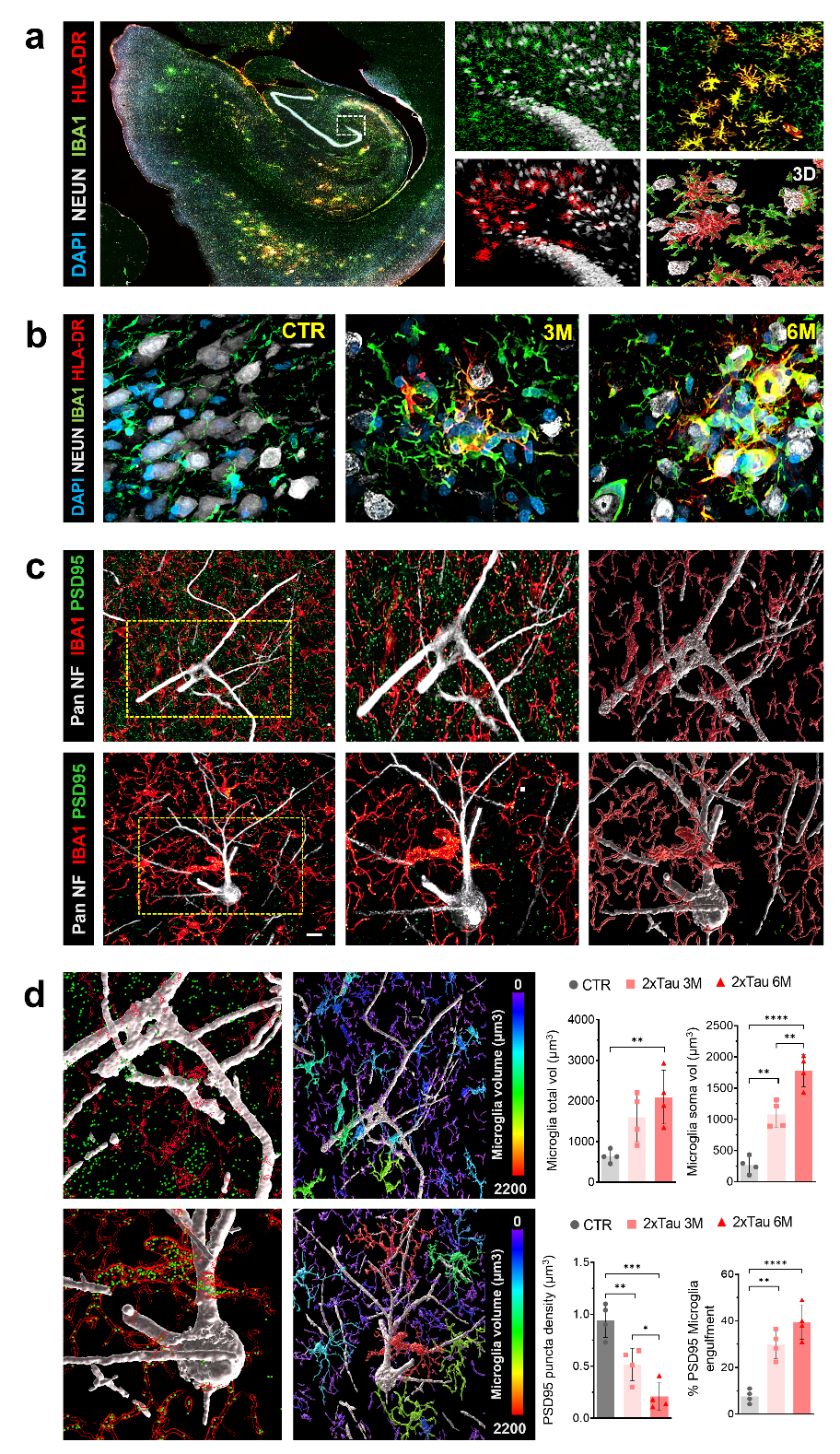
**Supplementary Figure 7 – Activated microglia is associated with synaptic and neuronal loss in the HF-ERC area.** (**a**) Quadruple labeling combining DAPI (blue), NEUN (white), IBA1 (green), and activated marker for microglia HLA-DR (red) was performed across vulnerable regions following the AAV-2xTau injection. A magnified micrograph in the CA3/Hilus region highlights the localized expression of HLA-Dr by a selective population of microglia (**b**). (**c**) Further investigation of microglial-activated profiles was performed in the ERC layer II, a major region involved in the initial stages of neuronal cell loss commonly observed in AD. Increased colocalization of IBA1 and HLA-DR was observed in microglia directly interacting with neurons (white) in this region. In addition, direct microglial contact induces synapse loss in vulnerable neurons in AAV-2xTau-treated animals, as shown by representative images in (**d**). Notably, a heat-map representation of the average microglia volume shows an increased volume of these cells in AAV-2xTau animals. 3D Confocal multilabel image acquisition and reconstruction allowed microglia total and soma volume analysis (µm^3^), as well as synaptic marker PSD95 general density (µm^3^) and direct engulfment by microglia (**d**). Scale bar: 10 µm, 15 µm (b), 10 µm (c), 20 µm. **p<.01, ***p<.001, ****p<0.0001, 2way ANOVA (b), One-way ANOVA (d), Tukey's post hoc test.


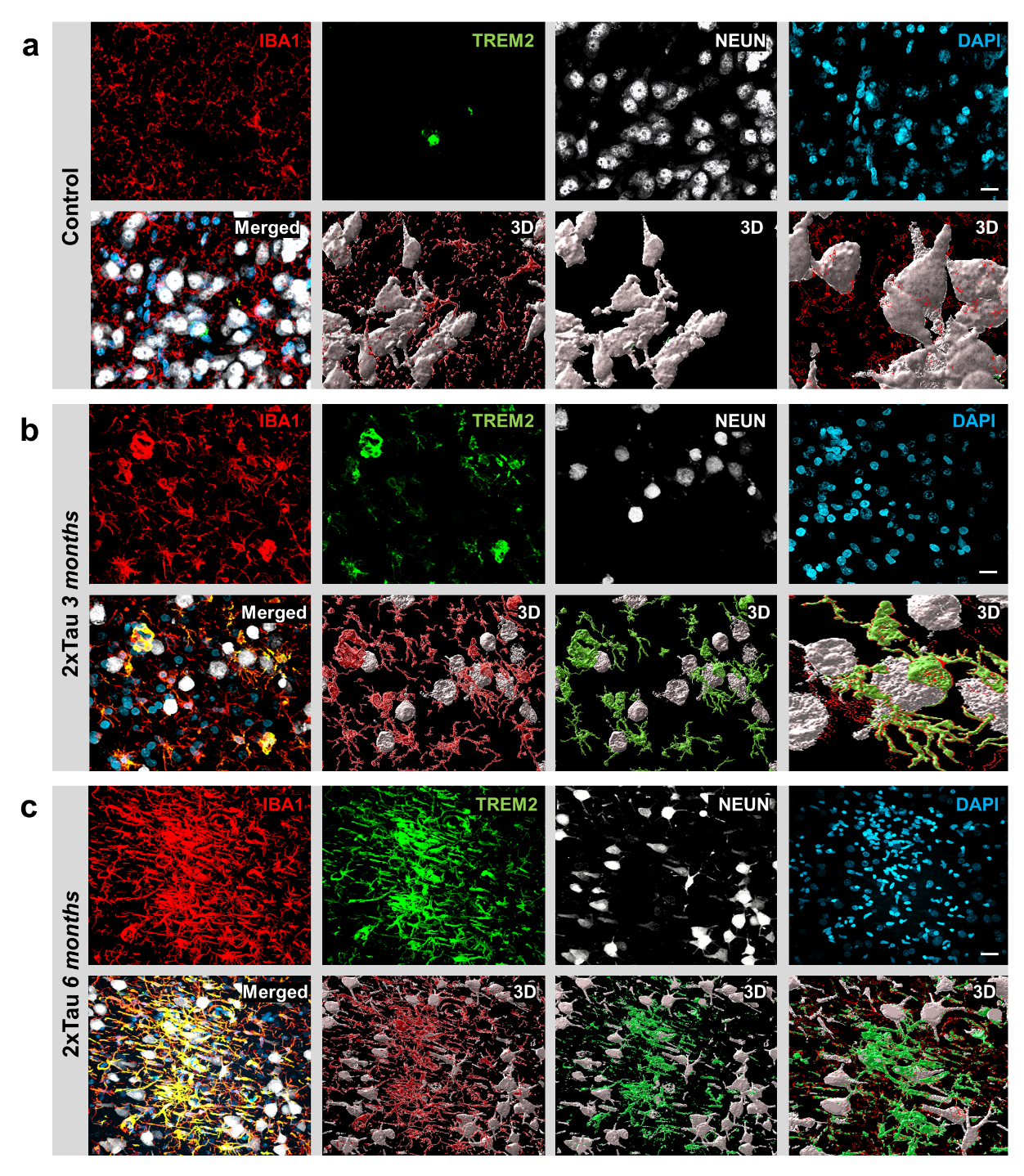


**Supplementary Figure 8 – Microglial TREM2 expression is time-dependent and varies between animals.** Separate channel visualization and 3D reconstructions of photomicrographs present in **Figure 8c** highlighting the expression of TREM2 (green) in microglia (IBA1 – red) and their spatial relationship to neurons (NEUN – white) and nuclei (DAPI – blue). In control animals (empty vector), minimal TREM2 expression is observed **(a)**. After three months of AAV-2xTau injections (AAV-2xTau 3M), TREM2 is co-expressed with IBA1 in ramified microglia, including neuron-contacting microglia, as illustrated in **(b)**. At 6 months following the injection (AAV-2xTau 6M), a higher expression of TREM2 is observed, often coinciding with densely packed microglial clusters (c). Some animals in the AAV-2xTau 6M group display extensive TREM2 expression associated with microglial infiltration and either the suppression of NEUN signal or neuronal displacement (d). Scale bar: 20 µm.


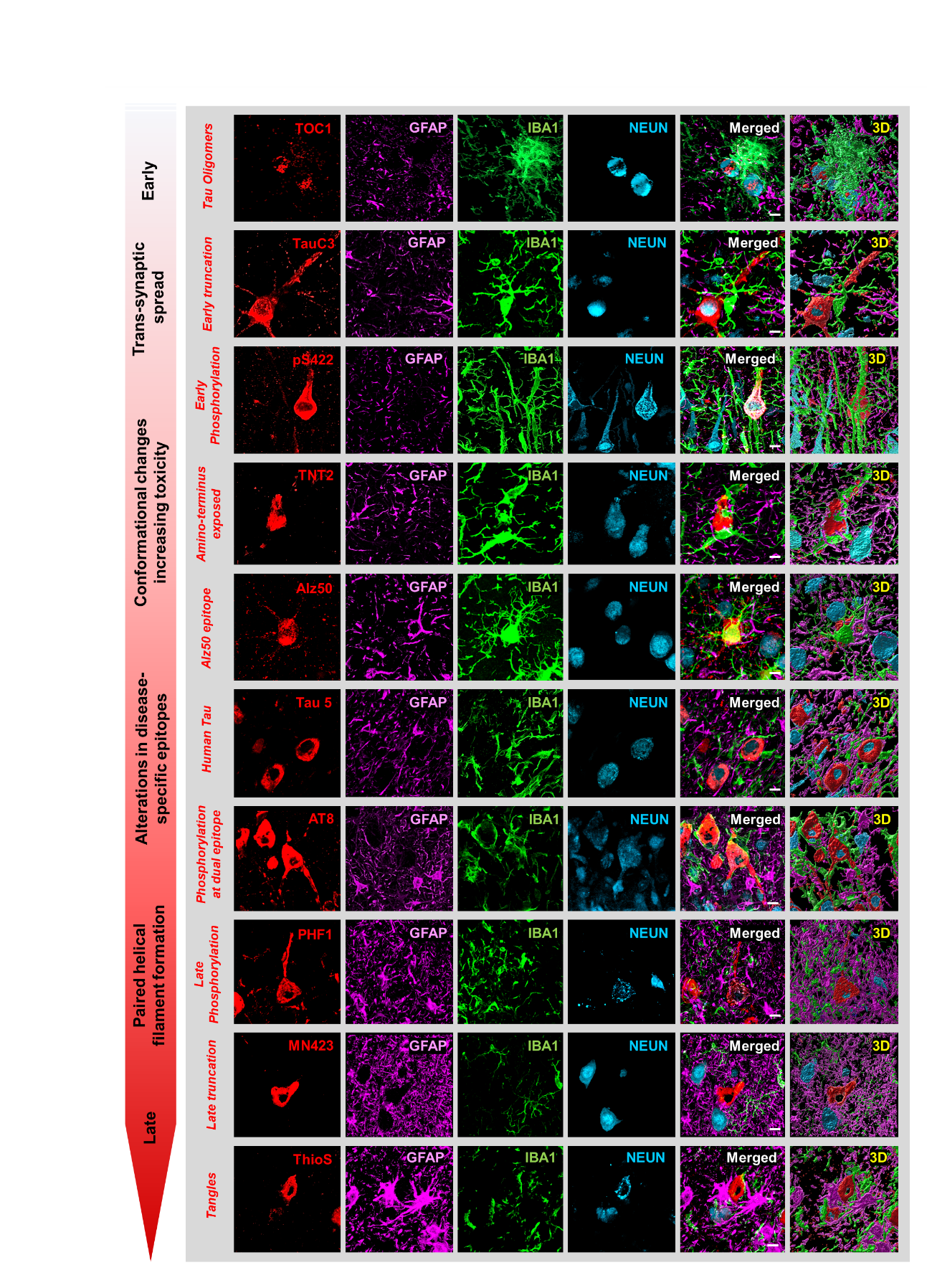


**Supplementary Figure 9 – Distinct Tau epitopes are associated with differential neuroimmune responses.** Separate channel visualization and 3D reconstructions of the photomicrographs in **Figure 9a** illustrate neuroimmune interactions in 3M and 6M AAV-2xTau animals. Separate channels correspond to neurons (NEUN - blue), microglia (IBA1 - green), astrocytes (GFAP - purple), and multiple Tau epitopes (red). Tau epitopes are organized from top to bottom based on their relative order of appearance and role played at distinct stages of Tau pathology, from earlier markers on the top to late-stage markers on the bottom, as indicated by the graphical elements on the left. This figure highlights the increased interaction between microglia (green) and the early markers of Tau pathology, while astrocytes (purple) become the predominant cell type associated with late Tau pathology markers. Scale bar: 5 µm.


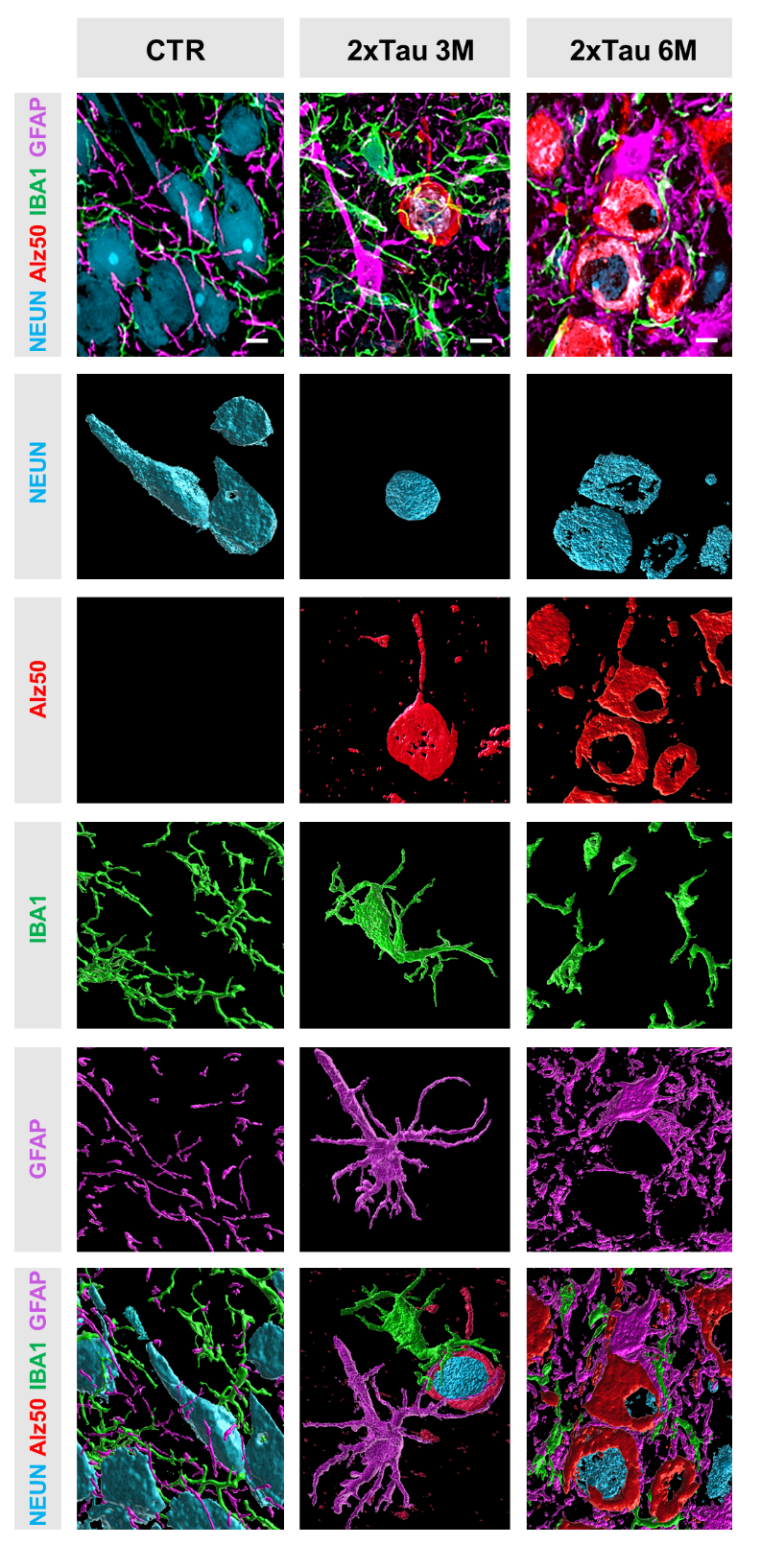


**Supplementary Figure 10 – Neuron-glia interactions shift following viral Tau induction.** Photomicrographs and 3D reconstructions from frontal brain sections of AAV-2xTau rhesus macaques subject to immunohistochemistry for the visualization of neurons (NeuN – blue), microglia (IBA1 – green), astrocytes (GFAP - magenta), and the pathology-associated Tau epitope Alz50 (red). In control animals (empty vector - CTR), microglia are predominantly ramified, and only thin, delicate GFAP+ processes are observed within neuronal fields. Three months after AAV-2xTau injections (2xTau 3M), enlarged microglia with few processes are observed in direct contact with Alz50+ neurons, in addition to astrocytic somas that are seldom observed in control cases. At six months (2xTau 6M), extensive astrogliosis is observed, with multiple swollen GFAP+ cell bodies and processes in areas containing Alz50+ neurons, while microglia have lost almost all processes. Scale bar: 5 µm.


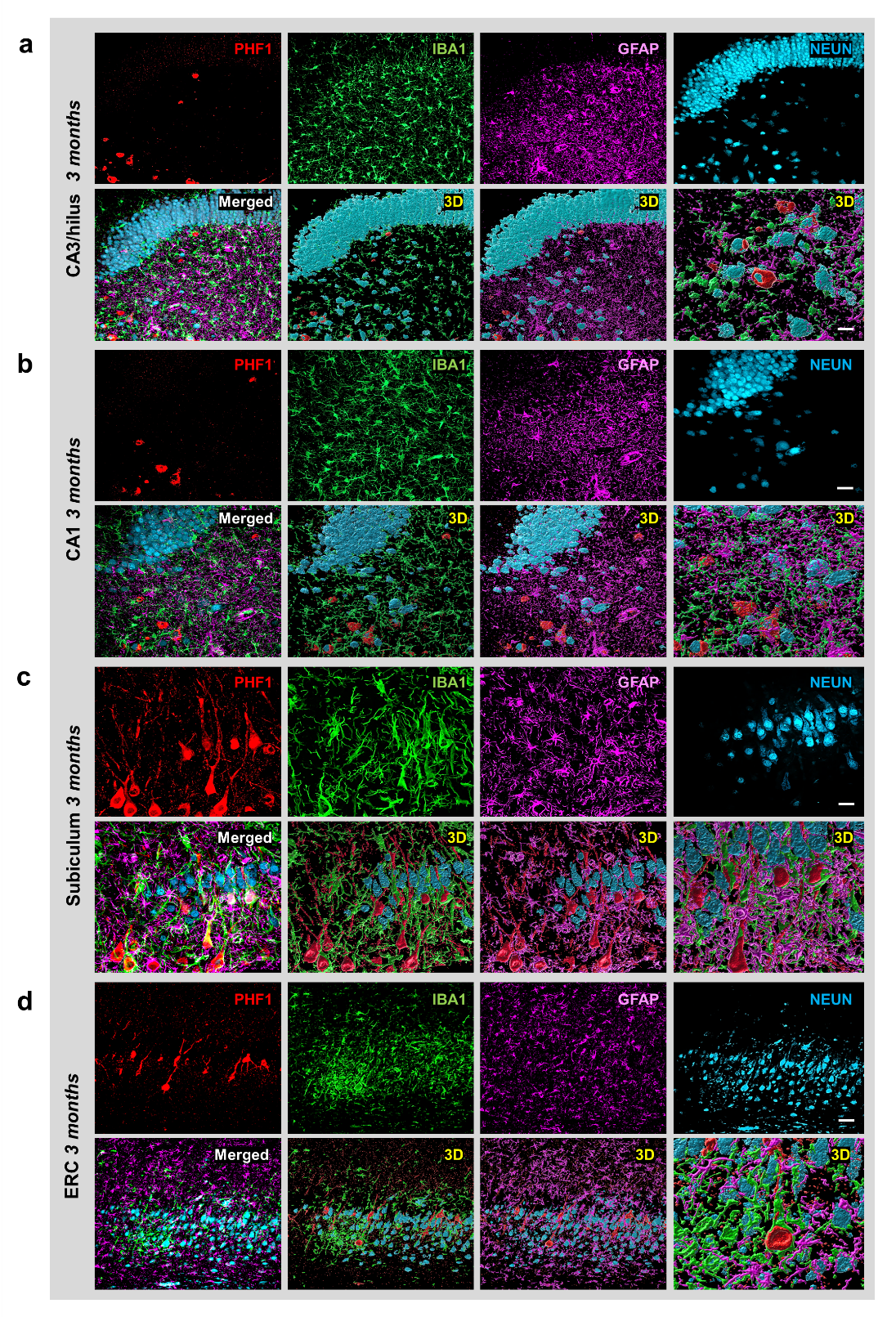


**Supplementary Figure 11 – Neuroimmune response to AAV-2xTau at 3 months.** Separate channel visualization and 3D reconstructions of the photomicrographs in **Figure 9b** from animals at 3 months after AAV-2xTau injection. Separate channels correspond to neurons (NEUN - blue), microglia (IBA1 - green), astrocytes (GFAP - purple), and the pathology-associated Tau epitope PHF1 (red) in the CA3/hilus **(a)**, CA1 **(b)**, subiculum **(c)**, and entorhinal cortex (ERC) **(d)**. Scale bar: 20 µm.


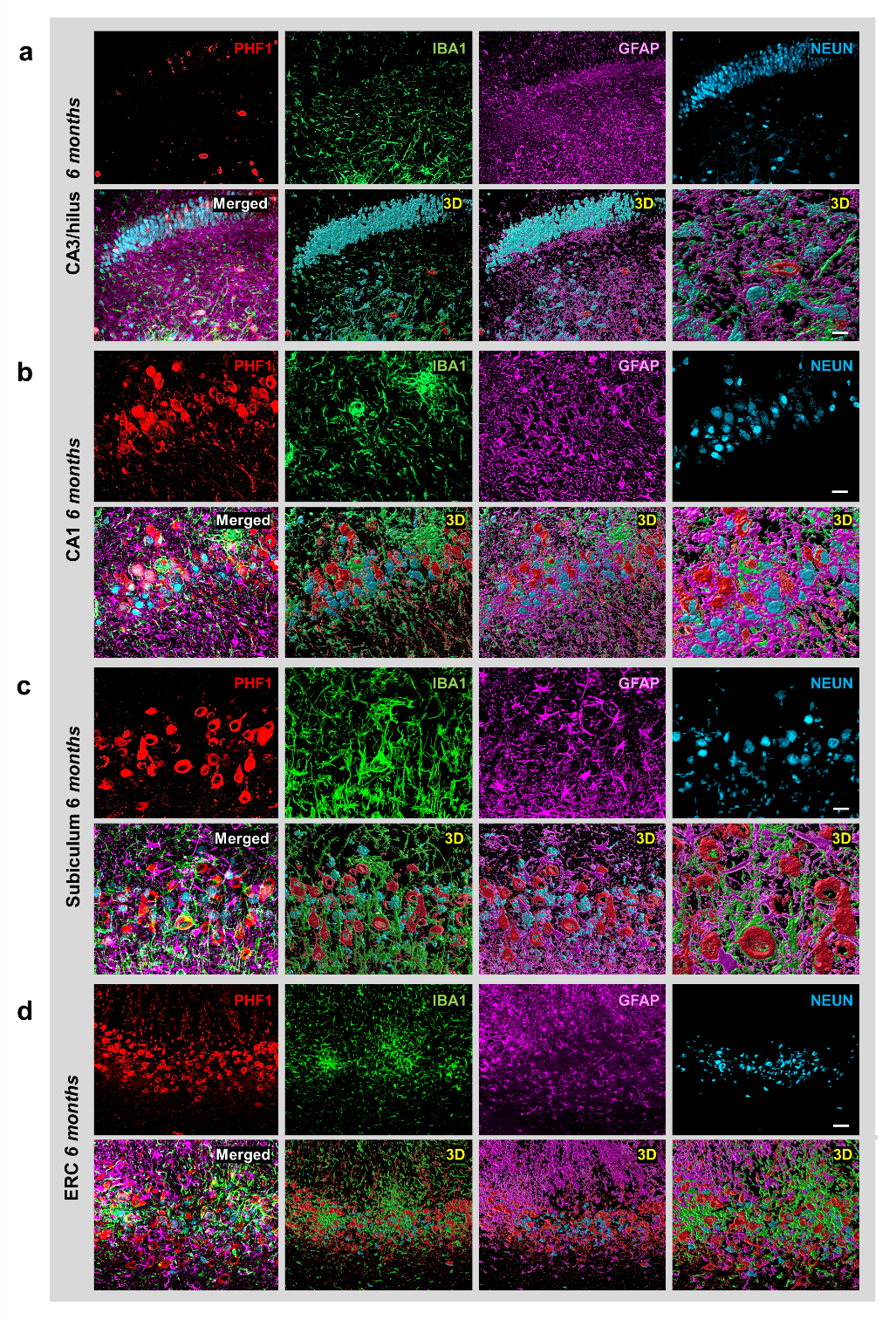


**Supplementary Figure 12 - Neuroimmune response to AAV-2xTau at 6 months.** Separate channel visualization and 3D reconstructions of the photomicrographs in **Figure 9b** from animals 6 months after AAV-2xTau injection. Separate channels correspond to neurons (NEUN - blue), microglia (IBA1 - green), astrocytes (GFAP - purple), and the pathology-associated Tau epitope PHF1 (red) in the CA3/hilus **(a)**, CA1 **(b)**, subiculum **(c)**, and entorhinal cortex (ERC) **(d)**. Compared to 3 months (Supplementary Figure S3), the extension of neuroinflammation in areas containing PHF-1+ neurons is visibly higher. Scale bar: 20 µm.


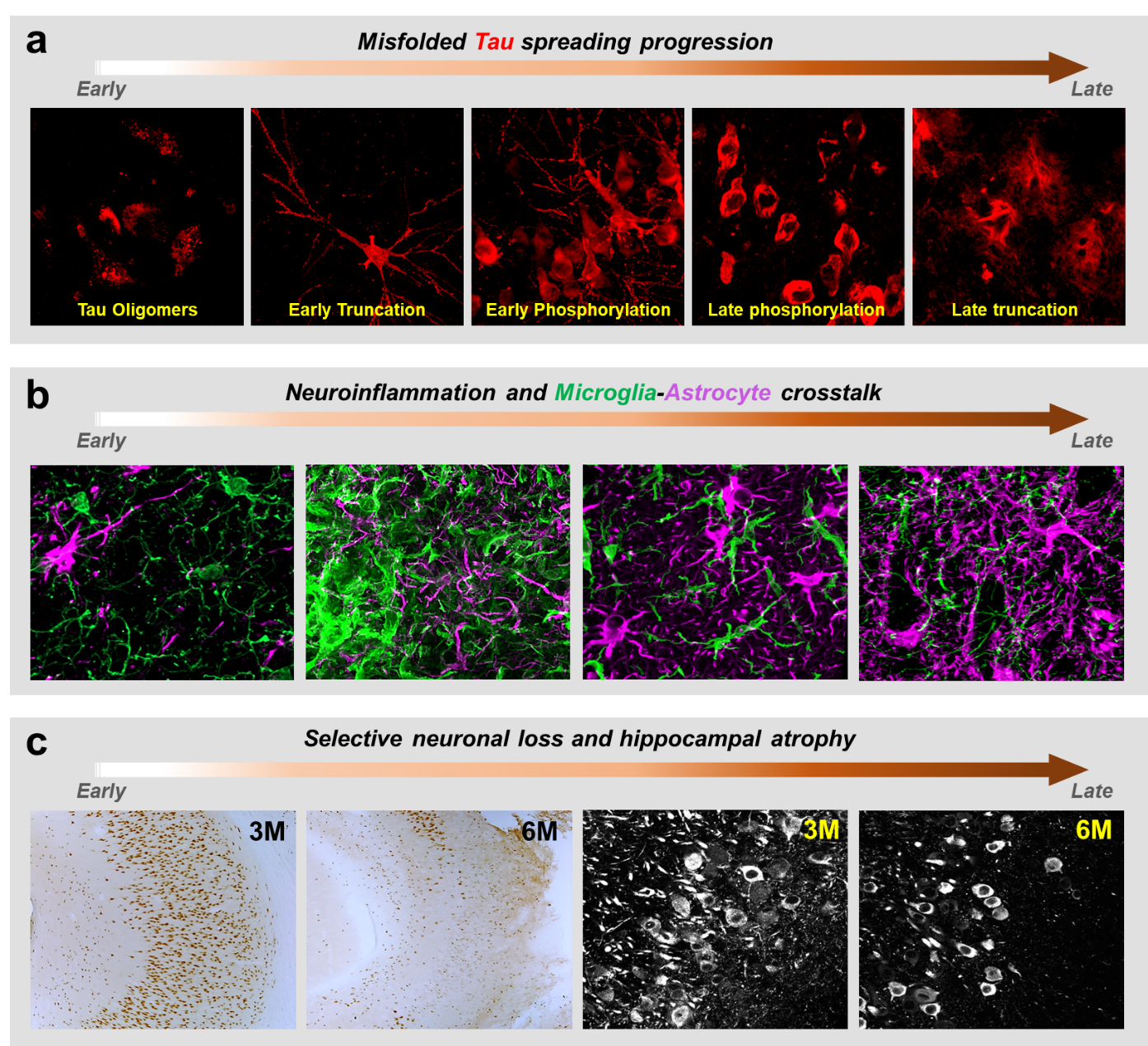


**Supplementary Figure 13 – Summary of the major findings observed in the hippocampal region of rhesus macaques following 3M and 6M after AAV-2xTau delivery in the entorhinal cortex.** A single delivery of an AAV containing a dual tau mutation (P301L/S320F) generates a temporal progression of AD-related pathology in vulnerable regions. Using a panel of markers targeting different stages of misfolded tau, we were able to unravel the different stages of pathological tau formation and propagation across the ERC-HF region (**a**). By combining this panel of tau markers with a microglial (IBA1, green) and an astrocytic marker (GFAP, purple), we uncovered how these glial cells interact in response to the presence and spreading of pathological tau (**b**). These biochemical alterations analyzed 3- and 6 months following injections help elucidate how neuronal cell death occurs in the primate brain and help clarify early pathological events observed in the brain of AD patients (**c**).
